# Mechanically interlocked molecular handcuffs

**DOI:** 10.1039/d2sc00568a

**Published:** 2022-03-16

**Authors:** Nicholas Pearce, Marysia Tarnowska, Nathan J. Andersen, Alexander Wahrhaftig-Lewis, Ben S. Pilgrim, Neil R. Champness

**Affiliations:** School of Chemistry, University of Birmingham Edgbaston Birmingham B15 2TT UK n.champness@bham.ac.uk; School of Chemistry, University of Nottingham, University Park Nottingham NG7 2RD UK ben.pilgrim@nottingham.ac.uk

## Abstract

The field of mechanically interlocked molecules that employ a handcuff component are reviewed. The variety of rotaxane and catenane structures that use the handcuff motif to interlock different components are discussed and a new nomenclature, distilling diverse terminologies to a single approach, is proposed. By unifying the interpretation of this class of molecules we identify new opportunities for employing this structural unit for new architectures.

## Abstract 2

Mechanically interlocked molecules that employ a handcuff component provide a pathway to highly unusual structures, a new nomenclature is proposed which helps to identify opportunities for employing this structural unit for new architectures.

## Graphical abstract

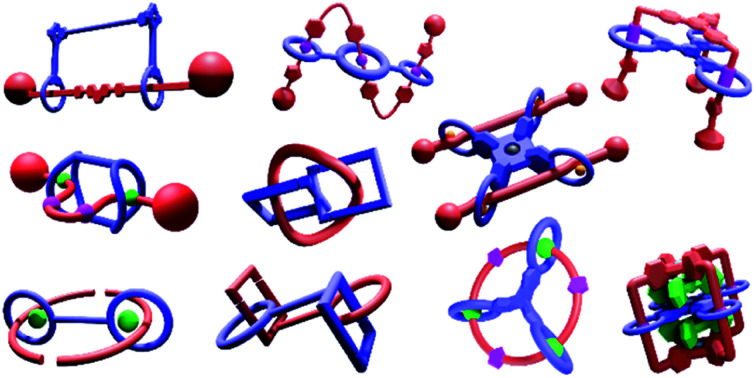

## 1. Introduction

The design of mechanically interlocked molecular components and architectures that resemble real-world objects is often a pursuit of the supramolecular chemist, as evidenced in the constructions of molecular knots,^1^ Borromean rings,^2^ and complex higher-order catenanes such as olympiadane which resembles the Olympic rings.^3^ The field has developed rapidly over the past few years and has recently been termed molecular nanotopology.^4^ Indeed, frequently when it comes to the nomenclature of such complex mechanically interlocked molecules, these structures have their names derived from the objects that their structure resembles. One example that fits this vernacular convention is molecular handcuffs, where the name perhaps implies their structure of a bis(macrocyclic) host ‘arresting’ a guest molecule.

In this review we seek to explore the diversity of structures based on molecular handcuffs and related systems, introducing the reader to these fascinating molecular structures and their utility in studying complex interactions. We also present a new convention for the naming of these intricate and complex structures. Whilst several attempts have been made to introduce a systematic nomenclature to the world of mechanically locked molecules,^5,6^ including recommendations by IUPAC,^5^ a widespread adoption of any naming convention has yet to occur. Although the name ‘molecular handcuff’ is open to interpretation, and at times even conflicts with other naming conventions, we hope in this review article to make some headway towards defining what qualifies as a molecular handcuff. In simplistic terms a handcuff molecule consists of two molecular components: a host component with a structure of two joined macrocycles; and a guest component that is threaded through each of the host's two rings, as a doubly mechanically interlocked molecule. In this simple sense the molecule resembles real-world handcuffs, which are two joined metal rings that can be used to restrain a prisoner. As with all areas of chemistry, our imagination is not restrained to the real-world and therefore the term molecular handcuff may be applied to ring systems of two or more joined loops, and molecular handcuffs may form multiple mechanical bonds with a single axle molecule.

**Fig. 1:**
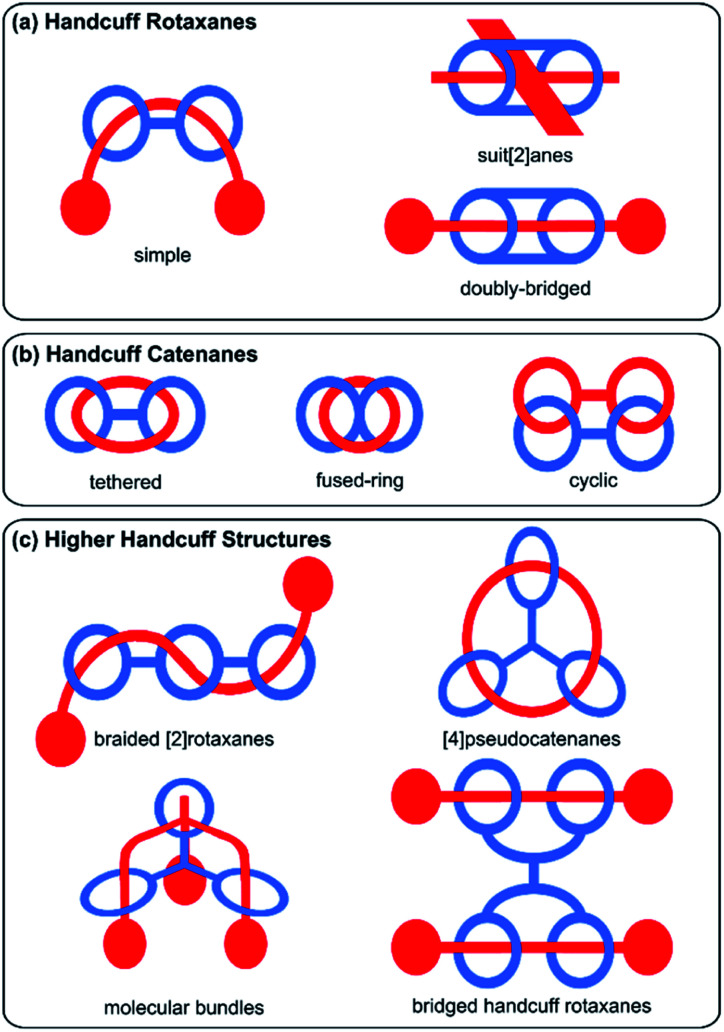
Schematic representation of the types of handcuff mechanomolecules reviewed in this work.

Broadly, mechanically interlocked molecules are classified into two main categories: catenanes and rotaxanes. Molecular handcuffs too, can be divided into these two classes. For handcuff rotaxanes, the guest ‘axle’ does not form a closed macrocyclic loop but should penetrate both host macrocycles (Fig. 1a). The analogy with real world handcuffs is clearest in the case of handcuff rotaxanes, where the handcuffs represent the bis(macrocyclic) species and the person's arms correspond to the threaded dumbbell molecule. In this physical analogy, the arrestee's wrists serve as the recognition sites for the handcuff clips. In the case of handcuff catenanes (Fig. 1b), a bis(macrocyclic) molecule is threaded through both of its macrocycles by another macrocyclic guest molecule. This guest species may assume the form of a bis(macrocycle) itself, so both bis(macrocycles) are mutually interlocked as a cyclic bis[2]catenane.

The diversity of mechanically interlocked molecules invokes topological and architectural considerations, especially when it comes to rationalising their structure and nomenclature. As such, we have chosen to omit certain examples from this review that do not follow either a topological or architectural approach to the goal of achieving a handcuff structure. Generally, those mechanically interlocked molecules that employ a host molecule with a cryptand or cage-like structure,^7,8^ have been excluded, since the cavity of these hosts typically presents only one guest recognition site and demonstrating that multiple rings have been threaded is debatable (or that multiple mechanical bonds are present) from a strictly topological standpoint. We have also chosen to omit unimolecular species such as pretzelanes,^9,10^ which resemble handcuffs that are ‘arresting’ themselves, as well as bis(macrocyclic) hosts that serve multiple guests, where the handcuff is serving as a tether between the two ensnared guests. We have, however, included higher order handcuff structures: species that comprise an oligo(macrocyclic) host to a single guest molecule that threads each of the host's macrocycles, since these species are analogous to handcuffs that would be used to arrest an entity with additional arms. These structures are clearly related to the other forms of molecular handcuffs, since in every case, the parent compound can be reduced to a handcuff system by rationalisation of the oligo(macrocyclic) host as a bis(macrocycle) that possesses additional complexity (Fig. 1c).

Considering these factors, this review aims to comprehensively survey the molecular design and synthesis of mechanically-bonded handcuff architectures as well as providing an insight into their properties and functions. We have framed our discussion of molecular handcuffs against other naming conventions, where relevant, to draw comparisons between the taxonomy of mechanically interlocked molecules, as well as to inform the reader of handcuff examples that have been alternatively categorized. We have used a new terminology to describe handcuffed structures of the form (*x*)handcuff [*y*]rotaxane/catenane. In this naming system *x* refers to the number of threaded macrocyclic hosts that are joined in the handcuff component of the mechanically interlocked molecule. Thus, if two macrocycles are joined, as in the simplest handcuff systems, then *x* = 2; if there are three joined macrocycles then *x* = 3 and so forth. If only two rings of a tris(macrocyclic) host are occupied by the guest, the result would be a (2)handcuff. The second term, *y*, refers to the number of mechanically bonded components of the rotaxane, or catenane, following the well-established protocols in the field.^5^ Examples discussed throughout this article will make it clear how this terminology has been applied and where additional descriptors are required. Our discussion of handcuff architectures has been divided into three main classes based upon their structure: (i) handcuff rotaxanes, (ii) handcuff catenanes and (iii) higher order handcuff architectures (Fig. 1), and each class will be discussed in turn.

## 2. Handcuff rotaxanes

Perhaps the most straightforward form of molecular handcuffs to rationalise are handcuff rotaxanes that consist of a bis(macrocylic) component that forms two mechanical bonds with a single axle component that has threaded through each macrocycle. Invoking our proposed nomenclature described above, such a system would be termed a (2)handcuff [2]rotaxane and is the closest parallel to ‘real-world’ handcuffs: the bis(macrocyclic) host reflects the pair of handcuffs and the molecular axle can be thought of as the person trapped within them. Indeed the most common form of molecular handcuffs are (2)handcuff [2]rotaxanes. An alternative name for this type of bridged macromolecule was proposed by Vögtle,^11^ who pioneered some of the early work into handcuff rotaxanes. He suggested ‘Bonnanes’ after the city of Bonn in which they were based, the former bridge capital of Germany, to reflect the bridge between the pair of macrocycles.

A unique opportunity afforded by handcuff rotaxanes that does not exist for simpler [2]rotaxanes is the possibility of mechanically bonding the system with a single stopper group that lies between both macrocycles. If the system is rigid enough, dethreading can still be prevented by a sufficiently bulky central group, without the need for the axle to possess stoppers at each terminus. This principle is well demonstrated by ‘suit[*n*]anes’, a term proposed by Stoddart^12^ to describe a molecular body encapsulated within a molecular suit in which *n* limbs are protruding. In the case of a suit[2]ane, two limbs of a central axle (the ‘torso’) protrude from a bis(macrocyclic) host that is typically connected with two covalent bridges (the ‘suit’). Due to the great similarity between suitanes and handcuff rotaxanes, we reason that suitanes are a form of handcuff rotaxane and will discuss them as part of this section.

### 2.1 Strategies for handcuff rotaxane synthesis

Much as there are different mechanisms by which [2]rotaxanes can be prepared (for example: capping, slipping, ring-clipping and templating), several strategies towards the synthesis of handcuff rotaxanes have been demonstrated that include the linking of macrocycles in a 2-ring [3]rotaxane; the linking of axles in a 2-axle [3]rotaxane; as well as the more traditional threading followed by stoppering method. Although to date the number of different synthetic approaches that have been realised in pursuit of handcuff rotaxane synthesis is quite small, we note that other methods are viable, including both ring-clipping and templating. For example, consider the case of ring-clipping; the precursor to the bis(macrocyclic) species might exist as a double clamp like molecule that associates with two active sites on a separate axle molecule; the handcuff rotaxane could then be generated by simultaneous ring closing reactions. Similarly, with suitably designed molecular components, concurrent active-templating reactions could be used to end-cap a central axle within the cavities of a bis(macrocylic) catalyst. Since these methods of handcuff synthesis have yet to be achieved, we will duly focus on those strategies which do have a literature precedent and describe them in more detail.

#### 2.1.1 Linking synthesis

One of the earliest examples of a handcuff rotaxane-like molecule came from the Stoddart group in 2005, who employed a dialkylammonium axle that formed a pseudo[3]rotaxane with two crown ether hosts bearing olefin groups, which could then be coupled by olefin cross metathesis to afford a pseudo(2)handcuff [2]rotaxane.^13^ Also in 2005 the Stoddart group reported a strategy for the reaction of dialdehyde and diamine compounds that are designed to clip around dialkyl-ammonium containing dumbbells, forming imine bonds under thermodynamic control.^14^ Amongst a number of examples, the authors described a ‘cyclic [4]rotaxane’ which contains two handcuff molecules but as each of these do not bind to the same axle component the molecule does not fit our definition of a mechanically interlocked handcuff structure.

**Fig. 2:**
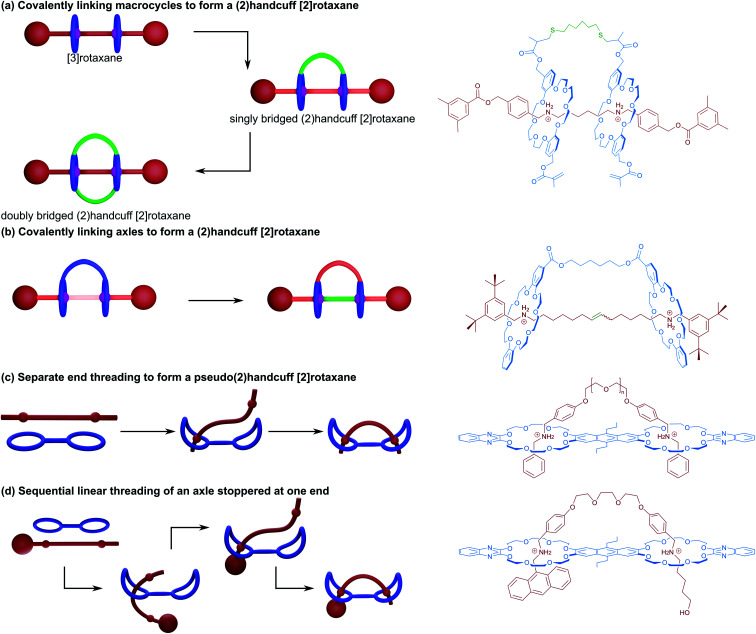
Methods of (2)handcuff [2]rotaxane synthesis. (a) Linking macrocycles; (b) linking axles; (c) separate end threading; and (d) leading end threading.

Soon afterwards, Sato and Takata also used the strategy of covalently linking the macrocycles of a [3]rotaxane to form a true (2)handcuff [2]rotaxane.^15^ Two dibenzo[24]crown-8 (DB24C8) macrocycles functionalised with methacrylate groups on each of the benzene rings were threaded onto a dialkylammonium axle and end-capped, producing a [3]rotaxane (Fig. 2a). Connection between the wheels was subsequently achieved by two conjugate additions of hexanedithiol to this [3]rotaxane, forming the singly bridged handcuff compound as the main product when using lower reactant concentration. When the initial concentration of reactants was increased, an additional tether between the remaining methacrylate groups on the other side of the macrocycles is able to react, forming a doubly bridged (2)handcuff [2]rotaxane. Interestingly, when the conjugate addition reaction was performed on free macrocycle no bis(macrocyclic) product was detected, instead only a polymer was observed, revealing both the advantages of organising reactive components with mechanical bonds and the control over the chemistry provided through a handcuffing strategy.

The related approach of threading two axles through each ring of a bis(macrocycle) prior to linking the axles together was exhibited by Iwamoto and co-workers.^16,17^ DB24C8 macrocycles were linked with ester groups and threaded with half-dumbbell alkylammonium axles bearing terminal olefins. The preorganisation of these components permitted linking of the axles with an olefin metathesis reaction, producing the handcuff rotaxane (Fig. 2b). Ultimately the authors were interested in pursuing a high yielding synthesis of a [3]rotaxane, which could be produced directly from the handcuff by using a saponification reaction to remove the bridge between the macrocycles. The rationale of temporarily tethering the macrocycles and proceeding *via* a handcuffed intermediate proved advantageous towards overcoming the entropic limitations of directly coupling two pseudo[2]rotaxanes, where the labile macrocycles could more easily slip away prior to the metathesis reaction.

#### 2.1.2 Threading synthesis

The majority of examples of (2)handcuff [2]rotaxanes have been formed by employing a threading and end capping process. Typically, in this procedure an axle molecule that bears two host recognition sites is mixed with a bis(macrocycle) and the two components are allowed to associate before both ends of the axle are reacted with sterically encumbering stopper groups. The recognition sites of each component must be at an appropriate spacing to permit the threading interactions, or the tethers linking each recognition unit ought to offer enough conformational flexibility such that a bimolecular association is possible. The mechanism of threading can vary between systems: the axle molecule may thread outwardly, with each end separately piercing each macrocycle (this is necessarily the case if the axle contains a central group too large to pass through either macrocycle); or by a leading end of the axle first threading one macrocycle, then the other.

A detailed study into the thermodynamic and kinetic effects of these different threading mechanisms in pseudo(2)handcuff [2]rotaxanes has been published by the Schalley group.^18^ For homodivalent (symmetrical) pseudorotaxanes utilising ammonium/crown ether recognition, two mechanistic steps are involved: (i) the threading of one ammonium arm of the axle into one crown ether, followed by (ii) the threading of the second arm into the remaining crown (Fig. 2c). The Schalley group showed that if a pseudo[3]rotaxane comprised of two ammonium axles and a bis(crown ether) is mixed with a pseudo[3]rotaxane made from a divalent ammonium axle and two crown ethers, the mixture will form an equilibrium that shifts towards the formation of the pseudo(2)handcuff [2]rotaxane product where the divalent ammonium axle is associated with the bis(crown ether), and the remaining monovalent components form pseudo[2]rotaxanes. Thus, for handcuff rotaxanes, the second threading event becomes favourable due to a chelate cooperativity. Furthermore, the effects on binding of the axle to the bis(macrocyclic) host can be pronounced by additional favourable interactions between the spacer groups of the host and guest entities. If the spacer is ‘non-innocent’ and participates in a binding interaction or is the correct length to modulate additional favourable interactions, an enhanced chelate cooperativity is observed, representing additional thermodynamic effects upon threading.

On the other hand, Schalley and co-workers^18^ were able to force sequential linear threading of a divalent ammonium axle that was stoppered at one end through a heterodivalent bis(crown ether) with two different cavity sizes. In this case threading now involves three steps: (i) threading of the leading end of the axle into the larger crown ether cavity up to the first ammonium recognition site, (ii) migration of the larger crown ether along the axle to the second ammonium station and lastly, (iii) the threading of the leading end of the axle into the smaller crown ether cavity (Fig. 2d). The different cavity sizes allowed for the kinetics of each threading step to be monitored independently, with (i) occurring on the millisecond timescale, (ii) on the second timescale and (iii) on the order of minutes. Differences in spacer length were also considered, the authors this time finding that longer spacers were more able to overcome the activation enthalpy of the second threading event, where strain in the transition state presents a barrier to handcuff formation. Consequently, the kinetically optimum spacer length may be different to the thermodynamically ideal option. Importantly, the authors conclude that whilst longer spacers can be slightly less favourable due to entropic effects, this effect is only slight, and that long, flexible tethers between binding sites remain potent to the assembly of handcuff species. A similar study into this sequential ‘inchworm’ mechanism of threading has been recently published by Chen and co-workers,^19^ also utilising multivalent ammonium axle/crown ether host binding interactions. Through a combination of NMR spectroscopy and molecular dynamics simulations, a study into the kinetics of threading the three station axle through either bis(macrocyclic) or tris(macrocyclic) hosts was performed. The results supported those of Schalley and co-workers, confirming that whilst the first threading event is very fast, it was considerably slower for the additional threading steps to occur, due to a gradual decrease in the freedom of movement of both host and guest components imposing a significant entropic effect upon the system. When a more conformationally flexible guest was used, complete threading was achieved more quickly, accompanied by an increase in the proportion of complexed components, establishing how important axle flexibility can be in the successful synthesis of handcuff rotaxanes.

### 2.2 Chemical topology in handcuff rotaxane systems

The arrangement of components within handcuff rotaxanes can lead to deeper structural complexity in the form of topological isomerism. This topoisomerism may become apparent in the relative orientation of the two macrocycles (especially if chiral or cone-shaped macrocycles are used), or in the way the axle weaves around the host before threading. For heterodivalent bis(macrocycles) and directional axles, the order in which each ring is threaded also affords the possibility of topoisomerism. In this section some of the ways in which the topology of handcuff rotaxanes has been studied will be explored.

#### 2.2.1 Stereoisomerism in handcuff rotaxanes

**Fig. 3:**
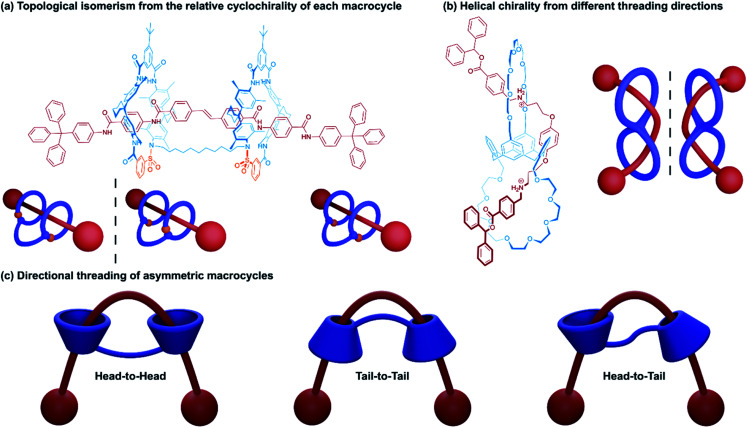
Topological isomerism in (2)handcuff [2]rotaxanes. (a) Relative cyclochirality of each macrocycle; (b) helical chirality resulting from direction of threading; (c) directional threading of asymmetric macrocycles.

One of the first reported syntheses of a (2)handcuff [2]rotaxane came from the Vögtle group in 2006 who were investigating covalent reactions of rotaxanes for further preparative chemistry.^11^ Vögtle and coworkers first prepared a [3]rotaxane that consisted of two sulfonamide containing macrocycles about a single axle (Fig. 3a). By reaction of the sulfonamide groups with a dihaloalkane tether, a covalent bridge was installed between the two macrocycles, forming a handcuff rotaxane by a linking synthesis. Due to the presence of one sulfonamide group per macrocyclic ring, the possibility of cyclodirectionality is introduced depending on the whether the sequence of sulfonamide and amide groups occurs in a clockwise or anticlockwise manner about the axle. Since an achiral axle was employed, cyclochirality^20^ is only manifested in the relative orientation of the two macrocycles, both in the parent [3]rotaxane and the handcuff rotaxane: a *meso* form of the handcuff exists where both macrocycles share the same orientation and a pair of enantiomers (d and l forms) arise if the macrocycles have opposite directionality. All three cyclodiastereoisomeric forms were detected and separation proved to be possible. The chirality of the parent [3]rotaxane was more pronounced in the CD spectra of the enantiomers than in the handcuff form where the wheels could not rotate freely with respect to each other due to the tether, a finding which may be of significance in the creation of molecular motors or ratchets where controlled rotational motion is essential.

A study by Tokunaga and co-workers^21^ examined the formation of a helically chiral (2)handcuff [2]rotaxane from achiral components. A calix[4]arene frame was used to orthogonally position two crown ether macrocycles, which were attached to alternating subunits of the calixarene. A bis-ammonium axle was complexed with the bis(calix-crown ether) and stoppered (Fig. 3b). Given the orthogonal orientation of the macrocycles, following the first ammonium/crown ether binding, the second threading event can occur from either side of the other macrocycle. Since threading from either direction is equally likely, a racemic mixture of the two helical handcuff enantiomers is produced, which could be separated by chiral HPLC. As with the cyclodirectional macrocycles of Vögtle's system, chirality was only induced when both wheels are threaded.

Tokunaga and co-workers also reported an alternative approach to introducing chirality to handcuff rotaxanes.^22^ In this later example a (2)handcuff [2]rotaxane was prepared using a handcuff molecule comprising two crown ethers linked through a biphenyl group combined with a symmetrical bis-ammonium salt. The resulting species exhibits both mechanically planar and axial chirality.

Another form of stereoisomerism in handcuff rotaxanes can be instituted using non-symmetrical macrocycles. Neri and coworkers note that for a bis(macrocycle) with cone-shaped calix[6]arene hosts, three stereoisomeric handcuff rotaxanes are possible with head-to-head, tail-to-tail and head-to-tail relative orientations of the calix-wheels (Fig. 3c).^23^ Taking advantage of the “*endo*-alkyl rule”,^24^ the preference for directional threading of a calixarene by an alkylbenzylammonium guest to have the benzene ring of the guest on the phenol rim side of the calixarene, Neri and coworkers were able to synthesise a (2)handcuff [2]rotaxane with exclusively tail-to-tail orientational isomerism. Knowledge of synthetic strategies such as this can be valuable in the construction of molecular architectures with higher-order topologies, without the concern of generating undesired mechanoisomers.

#### 2.2.2 Mechanical bond location

**Fig. 4:**
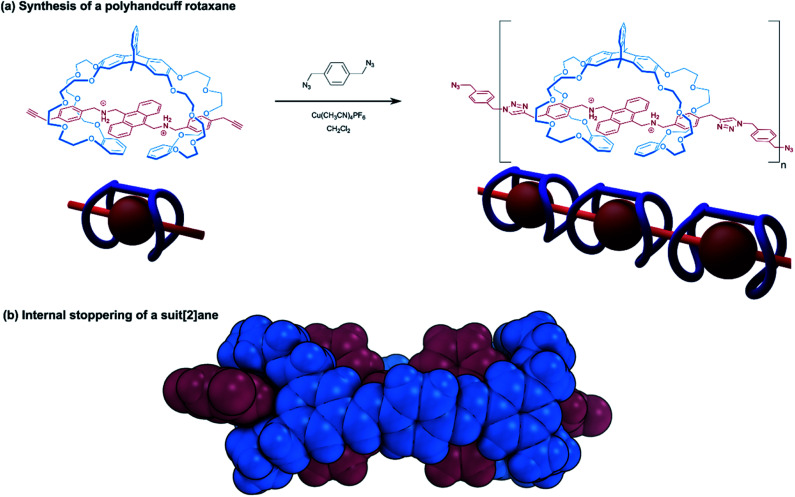
Handcuff rotaxanes with sterically limiting groups placed between the two macrocycles. (a) Synthesis of a polyhandcuff rotaxane; (b) single crystal X-ray structure of a suit[2]ane.

Described in the Introduction to this section, handcuff rotaxanes allow for the prospect of forming a mechanically bonded system where only one stopper group is required. Dethreading of the bis(macrocycle) can be limited if the axle molecule is sufficiently long that one of the macrocycles would have to pass over a sterically demanding blocking group located at the axle's centre. Indeed, this principle has been demonstrated by Chen and co-workers,^25^ who were able to synthesise a pseudo(2)handcuff [2]rotaxane by complexing a triptycene derived bis(crown ether) host and a dibenzylammonium guest that contained a central anthracenyl unit too large to pass through the crown cavity. Despite lacking terminal blocking groups on the threaded axle, the stability of the complex was confirmed by NMR and fluorescence spectroscopy, as well as through an X-ray crystal structure. The guest molecule was modified further to present terminal propargyl groups, for subsequent reaction of the handcuff complex. Instead of a reaction with bulky stoppering groups, Chen decided to combine the propargyl-handcuff with a diazide using a copper-catalysed azide–alkyne Huisgen cycloaddition or ‘click’ reaction to form a polyhandcuff rotaxane, averaging seven handcuff monomer units (Fig. 4a). To our knowledge, this is the only example of a polyhandcuff rotaxane, and it stands to reason that the bis(crown ether) hosts must be truly mechanically bonded since the axle is comprised of numerous repeating blocking anthracenyl moieties.

Stoddart introduced us to the concept of suitanes in 2006 ^12^ as a mechanically interlocked molecular architecture in which a central molecular body is encompassed by a close fitting molecular ‘suit’ that is distinguishable from capsules and carceplexes by the protruding limbs of the central body. In pursuit of this molecular composition, Stoddart and co-workers assembled a pseudo[3]rotaxane from two [24]crown-8 rings bearing aldehyde groups and a dibenzylammonium axle. The ammonium recognition sites of the axle were separated by a bis(anthracenyl) core, able to prohibit further movement of the macrocycles. A dynamic covalent chemistry approach was used to connect the two macrocycles in the pseudo[3]rotaxane with imine bonds through the reaction of the aldehyde groups with two equivalents of *p*-phenylenediamine, forming the suitane. Computational force-field modelling, coupled with solid-state structural data of the suitane from single crystal X-ray crystallography indicate that supplementary interactions between the phenylene linker and the anthracenyl core play a role in further stabilising the structure (Fig. 4b). Moreover, the integrity of the suitane's mechanical bonds was evidenced by the lack of any free host or guest present in a sample of the suitane that had been heated for 30 days. Comparing the suitane's synthetic strategy with the linking synthesis of Sato and Takata^15^ (see Section 2.1.1), reveals many similarities between the suitane and doubly-bridged handcuff rotaxanes: the difference in mechanical bond location is somewhat superficial to the overall structure; the deeper principles of rational molecular design and supramolecular chemistry unite suitanes and handcuffs.

In suitanes and doubly-bridged handcuff rotaxanes, the second connecting bridge between the macrocycles creates a third macrocyclic unit, the aperture of which lies perpendicular to those of the original macrocycles. The different composition of the third macrocycle endows it with different chemical properties to the two flanking macrocycles and thus, may provide the possibility of different host–guest chemistry. The threading modes of a macrotricyclic host consisting of two DB24C8 macrocycles, wherein one pair of benzene rings formed part of a triptycene bridge and the other pair an anthracene bridge, was examined by Chen and co-workers.^26^ The aliphatic paraquat derivative, 1,1′-di(6-hydroxyhexyl)-4,4′-bipyridinium formed complexes with the macrotricyclic host where each hydroxyhexyl tail participated in a threading interaction with each DB24C8 host, and could later be stoppered to form the corresponding (2)handcuff [2]rotaxane. Modification of the viologen to replace the hydroxyhexyl groups with a bulkier substituent that could not thread the crown cavity still allowed for construction of a [2]rotaxane, in this case however, it was the third macrocylic cavity, circumscribed by the triptycene and anthracene bridges that was threaded, and no handcuff was formed. A related structure published by Chiu and co-workers,^27^ complexed a bridged DB24C8 macrotricyclic cage with an alkylammonium axle to form a [2]rotaxane. The axle only contained one ammonium recognition site and consequently only one crown ether was threaded. Despite certain topological similarities between this rotaxane and handcuff structures, from a supramolecular standpoint, the mechanical bond location sets this structure apart from true handcuff species.

A conceptually related strategy has been reported recently by Pöthig and co-workers.^28^ In their system they assemble a [2]rotaxane using a macrocyclic component based on a so-called ‘pillarplex’, a complex in which two imidazolium containing macrocycles are linked through the formation of bis-N-heterocyclic carbene silver(i) moieties. This cylindrical macrocycle is threaded by simple a diamine rod which can be stoppered through amide bond formation. Interestingly, the silver cations can be removed by addition of acid leading to the formation of a [3]rotaxane in which the two imidazolium-containing macrocycles are separated from each other. The process is reversible establishing a mechanism for conversion between [2] and [3]rotaxanes. The system has clear parallels to the suitane structures described above.

### 2.3 Controlling motion in handcuff rotaxanes

Precise control over molecular motion is one of the most significant attributes of mechanically interlocked supramolecular chemistry. Compared to covalent chemistry, the freedom of movement permitted by the mechanical bond allows for the construction of more advanced molecular machines.^29^ In the field of rotaxanes, perhaps the most prevalent example of controlling molecular motion leading to a machine-like application is the construction of bistable molecular switches.^30^ The position of the macrocycle along an axle containing two chemically distinct stations can be reversibly controlled by external stimuli that favour interactions between one station over the other. In [2]rotaxane switches, typically the motion of the components is purely relative: the ring moving along the axle is indistinguishable from the axle moving through the ring. This is not necessarily the case in switches built using handcuff rotaxanes, where conformational rigidity of either the bis(macrocyclic) component or the axle means that more substantial rearrangement might be imposed on just one of the molecular species for the system to move between its two states. We will demonstrate this principle in detail by first considering motion in switchable (2)handcuff [2]rotaxane systems built around conformationally restrictive bis(macrocycles).

#### 2.3.1 Rigidity in the bis(macrocyclic) component

**Fig. 5:**
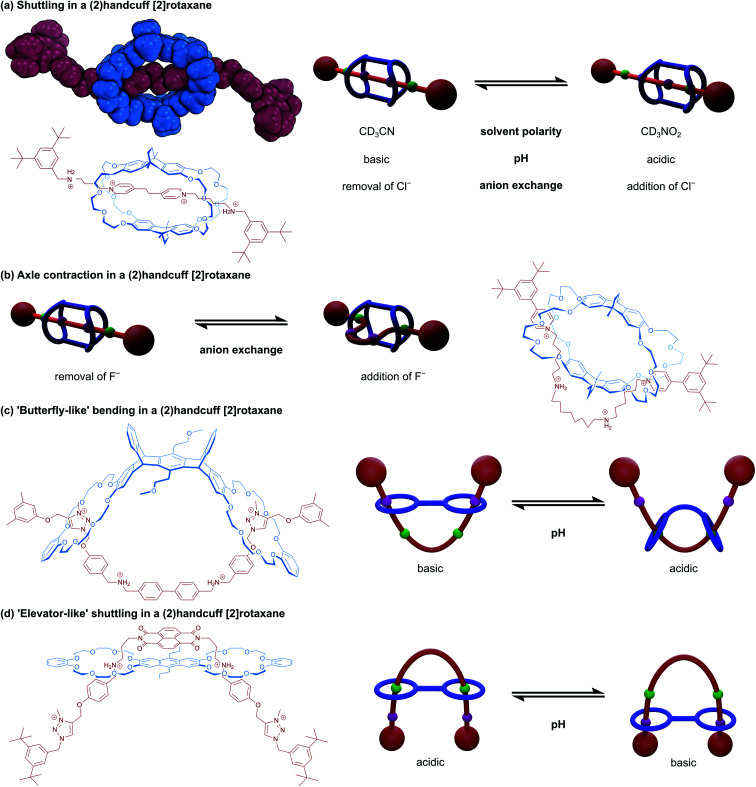
Controllable motion in (2)handcuff [2]rotaxanes. (a) Shuttling motion including a view of the single crystal X-ray structure; (b) axle contraction; (c) ‘butterfly-like’ bending; (d) ‘elevator-like’ shuttling.

An early doubly-bridged handcuff rotaxane was reported in 2007 by Chiu and co-workers^31^ that could undergo translational motion of the host about the axle in response to various stimuli. The system incorporated a macrotricyclic host; where two DB24C8 moieties were bridged with bicyclo[2.2.2]octyl linkers to form a rigid cage-like structure; and an axle with central pyridinium sites and terminal benzylammonium stoppers (Fig. 5a). Translation of the host to one end of the axle could be achieved by increasing affinity of the host for the ammonium stations, which proved to be possible through three mechanisms: (i) altering the polarity of the solvent, (ii) changing the counterions, or by (iii) lowering the pH. Decreasing the affinity of the host for the ammonium stations (*e.g.*, by increasing the pH), saw the position of the host return to the centre of the axle and occupy both pyridinium stations.

A subsequent collaboration between Chao and Chiu^32^ used the same macrotricyclic host with a restructured axle that contained central alkylammonium stations and pyridinium stoppers. Synthesised and crystallised as the PF_6_^−^ salt, the crown ethers interact with the central alkylammonium stations, and the axle can adopt a linear ‘stretched’ conformation (Fig. 5b). Upon the addition of four equivalents of tetrabutylammonium fluoride, the axle rearranges to allow for interaction between the pyridyl and crown ether groups, established by changes to the NMR spectrum of the handcuff that showed the extrusion of the central aliphatic chain through the flanking macrocycles with changes to the shifts of the pyridinium and aliphatic chain protons, indicating complexation. The authors advocate that this rearrangement is due to disruption of hydrogen bonds between the ammonium centres and crown ethers by the fluoride ions, leading to a more favourable interaction with the pyridinium units. As the fluoride salt, the axle of the handcuff is considerably shorter end-to-end since the central portion must move out through the aperture of the remaining (non-crown) ring, dubbed the ‘contracted’ form. The rigidity of the bridges connecting the crown ethers prohibits expansion of the host to accommodate this change. The length change between stretched and contracted states was calculated through molecular dynamics simulations as 36%, which is greater than in human muscle. Precipitation of the fluoride with calcium tetrafluoroborate restored the stretched conformation of the handcuff.

A very similar study into a handcuff rotaxane derived molecular muscles was published by Qu and co-workers.^33^ An anthracene bridged bis(crown ether) was complexed with an axle containing a central ferrocene unit, that was flanked by benzylammonium groups and capped with triazolium based stoppers. Under acidic conditions, the protonated ammonium groups interact strongly with the crown ethers and under basic conditions, the outmost triazolium moieties are preferred over the deprotonated benzylamines. Here, a length change of 15.8 Å or 48% is reported, facilitated by mutual rotation of the two cyclopentadienyl rings of the ferrocene, displaying a combination of both translational and rotary motion in a single-molecule system. Chen and co-workers also employed an axle composed of central benzylammonium recognition sites and flanking triazolium stoppers in an acid/base switchable (2)handcuff [2]rotaxane.^34^ Some key differences are that the DB24C8 macrocycles were connected as part of a larger, pentiptycene based scaffold, and secondly the central benzylammonium sites were directly adjoined as *p*-biphenyl (Fig. 5c). In this instance, although switching from ammonium to triazolium stations does cause a similar bending of the axle, a concomitant distortion of the pentiptycene host is required. This opening out of the bis(macrocycle) was likened to that of “a butterfly spreading its wings” and represents another form of controllable motion available to handcuff systems.

Elevator-like motion has also been described for handcuff rotaxanes. A two station benzylammonium/triazolium axle with a central naphthalene diimide (NDI) was complexed with an anthracene bridged bis(crown ether) by Liu and co-workers (Fig. 5d).^35^ At low pH, the inner benzylammonium groups are protonated and interact with the crown ethers, which also brings the axle's NDI and host's anthracenyl chromophores in close proximity (3.9 Å), quenching the fluorescence of the system. A charge transfer band can be observed in the absorption spectrum of the handcuff that is not present for either isolated component. Addition of base to the handcuff moves the bis(crown ether) elevator platform to the triazolium stations and separates the anthracenyl and NDI chromophores to a distance of 12.4 Å. Interestingly, we note that although Liu^35^ used the same bis(macrocycle) as Qu,^33^ the computationally calculated structures of the systems exhibit markedly different conformations of the bis(macrocycle). In Liu's work, the bis(macrocycle) possesses an almost planar conformation, which means the axle must adopt a U-shaped conformation to thread through both rings, and the two arms of the axle serve as the elevator ‘shaft’. On the other hand, in the system described by Qu, the crown ether units curl round and allow the axle to thread both rings whilst remaining roughly linear and stretched out. We reason that the size and charge complementarity between the two central components in Liu's handcuff may account for the conformational differences that lead to the dissimilarities in reported motion.

#### 2.3.2 Rigidity in the threaded component

**Fig. 6:**
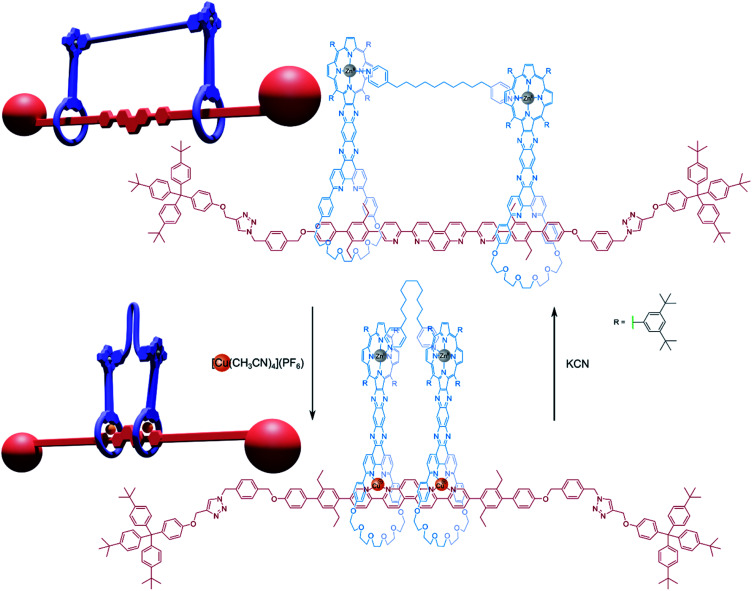
Compressor/extensor or accordion-like motion about a rigid axle.

When a more conformationally restrictive axle is used in a handcuff rotaxane system, it is instead the bis(macrocyclic) element that may have to endure structural rearrangement to allow for motion between stations along the axle. Sauvage and co-workers^36,37^ prepared a [3]rotaxane that used a linear rod-like axle whose central portion contained eight *para*-connected aromatic rings (Fig. 6). The central four rings provided a dual 2,2-bipyridine functionality from a 4,7-phenanthroline nucleus, allowing metal-templated complexation with 1,10-phenanthroline-based macrocycles, and the system was then stoppered using a ‘click’ reaction that introduced triazole stations to the axle as well as the encumbering end caps. The macrocycles of the [3]rotaxane also bore zinc-porphyrin functional groups that were connected directly to the phenanthroline moieties by a fused ring arrangement.

This [3]rotaxane could be converted into a handcuff rotaxane by using a linking synthesis to bridge the zinc-porphyrin centres with various dipyridyl ligands. When the rotaxane is complexed with copper(i), the positions of the two porphyrin plates are controlled by the geometry of the central axle, which forms coordination complexes with through its bipyridyl units with the phenanthroline groups of each macrocycle. Since the position of the macrocycles is rigidly defined, the linking dipyridyl tethering molecule must adopt a conformation to fit in the available space between the two porphyrin plates and may have to fold or compress considerably. If the copper(i) centres are removed, the macrocycles become free to glide along the 80 Å axle and the tether can extend to a preferred conformation that will instead determine the separation of the macrocyclic ‘wheels’. Sauvage found that this [3]rotaxane architecture could form handcuff compounds with tethers ranging from 2.8 Å (DABCO) to 18 Å (1,10-di(4-pyridyl)decane). In the case of the 18 Å tether, the metalated form of the handcuff complex becomes destabilised due to distortions imposed on the tethering molecule, however, the tether is still able to bind. As such, the authors rationalised the motion exhibited by this structure as functioning as a reversible ‘compressor/extensor’, or perhaps like an accordion.

#### 2.3.3 Redox control over handcuff systems

Mechanically binding molecules together as handcuffs is an efficient strategy for the positioning interesting molecular species close in space, whilst still allowing for free movement as required. Schalley and co-workers^38^ prepared a (2)handcuff [2]rotaxane from a tetrathiafulvalene (TTF) based bis(macrocycle) and an NDI equipped with the appropriate benzylammonium recognition sites. The absorption spectrum of the resulting handcuff displayed a strong intramolecular charge-transfer between the electron donating TTF unit and the electron accepting NDI, which may have helped the self-assembly process. Electrochemical investigations of the NDI-TTF handcuff revealed that the compound was stable across five redox states, and this redox stability was improved greatly over the free axle and wheel components, although the first oxidation potential (associated with the TTF chromophore) occurred at higher potential, due to stabilisation of the neutral state by intramolecular charge-transfer and the already dicationic nature of the handcuff. The optimised geometries of all five redox states of the complex were computed, finding that the separation between the NDI and TTF planes increases with both oxidation and reduction as the attractive charge-transfer interaction is lost.

Champness and co-workers^39^ also prepared redox active handcuff rotaxanes that positioned perylene diimide (PDI) chromophores in close proximity by means of pillar[5]arene/imidazolium rotaxane formation. The PDI units of axle and host species communicate strongly with π-stacking interactions, especially visible in the emission spectrum of the handcuff, which displayed an excimer-like profile, indicating electronic coupling between the two π-systems. This handcuffed compound was also stable across multiple redox states and capable of accepting four electrons. Spectroelectrochemical measurements of the two-electron reduced PDI–PDI handcuff unveiled a unique absorption profile for a PDI-based molecule that was assigned to the formation of a π-dimer that is not seen for rigidly spaced PDI–PDI dimers. The addition of two more electrons to the handcuff resulted in the loss of interaction between the two PDI units. A similar NDI–PDI handcuff rotaxane was also synthesised in which the addition of four electrons could be achieved in independent steps. Although features in the absorption and EPR spectra of the one-electron reduced NDI–PDI handcuff show little evidence of charge hopping between the two chromophores, this does seem to be possible for the two-electron reduced handcuff: EPR activity is lost, and the absorption profile is distinct from those of typical NDI^−^ and PDI^−^ chromophores. The strategy of molecular handcuffs provided a unique opportunity to probe interactions between redox centres that was enhanced by the flexibility granted to the system from the use of mechanical, not covalent bonds.

## 3. Handcuff catenanes

The natural partner to handcuff rotaxanes are handcuff catenanes, where the bis(macrocyclic) species is threaded through each ring by another single macrocycle. Following our suggested terminology, this arrangement would be called a (2)handcuff [2]catenane, reflecting that a two-ringed host is threaded by a second guest molecule. Since a handcuff catenane is the topological result of covalently connecting the two outer rings in a linear [3]catenane, the name pseudo[3]catenanes, introduced by Becher^40^ is also used occasionally in the literature. Interestingly, despite being a viable synthetic strategy for the synthesis of handcuff catenanes, we have not found any examples of handcuff catenanes that were constructed in this manner.

We also consider the mutual interlocking of two separate bis(macrocyclic) species to be a form of (2)handcuff [2]catenane: a two-ringed component is threaded through each macrocycle by a single species (that happens to also have bis(macrocyclic) structure), and two mechanical bonds are present in the form of the intertwined rings of a catenane, so the structure must qualify as both a (2)handcuff and a [2]catenane. This molecular architecture is most commonly referred to as a cyclic bis[2]catenane and we expand our discussion of handcuff catenanes to include these interesting arrangements.

### 3.1 Linear handcuff catenanes

Much as described for handcuff rotaxanes in Section 2.1, one might imagine different synthetic routes could be employed to produce a handcuff catenane. The catenane counterpart to the rotaxane ‘linking synthesis’ would involve covalently bridging two macrocycles in a [3]catenane to form the bis(macrocycle); whereas a threading synthesis would proceed as it does for a handcuff rotaxane, but instead of introducing stopper groups to the pseudo(2)handcuff [2]catenane, the ends of the threaded component are joined to each other, forming the third macrocycle. The latter case of threading a bis(macrocycle) is considerably more common amongst literature examples, though some differences arise from the connectivity of the two macrocycles. The two rings might be fused together by a central molecular species that serves as the recognition site for the thread, such that encircling this central species is accompanied by a concomitant threading of both macrocycles. Alternatively, the two macrocycles might be separated by a non-interacting tether and it is more important for the threading species to have affinity for the macrocyclic loops to ensure that threading occurs prior to the macrocyclisation step.

#### 3.1.1 Fused-ring handcuff catenanes

**Fig. 7:**
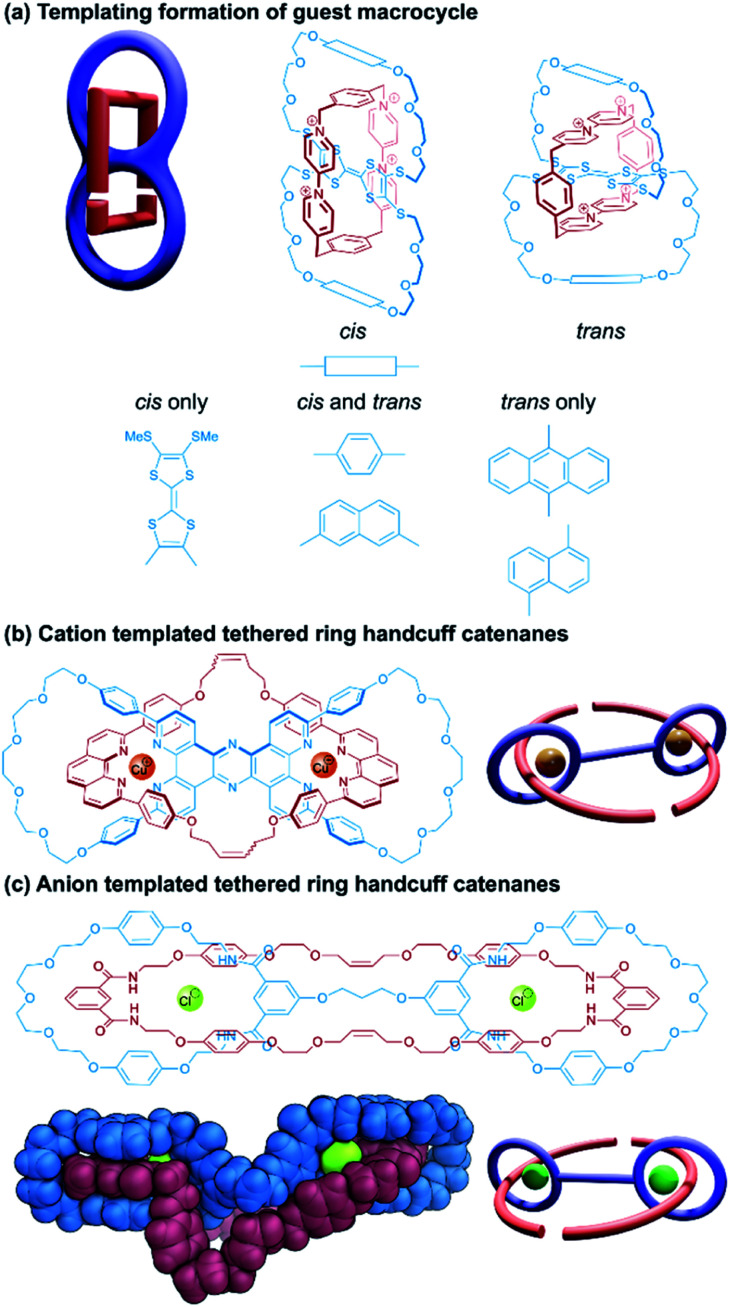
Structures of (2)handcuff [2]catenanes. (a) Fused-ring catenanes synthesised by templating the formation of the guest macrocycle; (b) tethered-ring handcuff catenanes synthesised through copper(i)-phenanthroline templating; (c) tethered-ring handcuff catenanes synthesised through chloride-amide hydrogen bond templating including a view of the single crystal X-ray structure.

The earliest synthesis of a (2)handcuff [2]catenane was reported by Becher and co-workers in 1995.^40^ Taking advantage of the tendency of TTF to form a strongly associated 1 : 1 charge-transfer complex with cyclobis(paraquat-*p*-phenylene), a bis(macrocycle) was designed around the TTF unit that would maximise π–π interactions with the paraquat species (Fig. 7a). Thus, macrocycles were formed by equipping a tetrathiolated TTF with glycolic loops, bridged by hydroquinone or anthraquinone spacers, that would lead to a separation between the TTF and these electron rich spacers of approximately 7 Å, optimal for binding the paraquat cyclophane. Unlike Schalley's TTF-based bis(macrocycle)^38^ where each end of the TTF unit was part of a separate macrocycle, in Becher's system, the macrocycles were connected across the long axis of the TTF centre, leading to *cis*/*trans* isomers that could interconvert in solution. Handcuff catenanes were synthesised *via* the reaction of the precursor components of cyclobis(paraquat-*p*-phenylene) in the presence of the TTF bis(macrocycle). For the hydroquinone-spaced bis(macrocycle), both *cis* and *trans* isomers of the handcuff were obtained, which could be separated from each other and characterised independently. These isomers did not interconvert, even in the presence of trifluoroacetic acid, indicating that the paraquat cyclophane inhibits TTF protonation that would lead to isomerisation. The oxidation potentials of the TTF centre increase for the handcuff catenanes compared to the free bis(macrocycles) due to charge transfer between the cyclobis(paraquat-*p*-phenylene) and the encircled TTF, though no significant difference was noted for the *cis* and *trans* isomers, implying that the change in handcuff configuration does not greatly affect its electron donating ability.

The handcuff catenane containing anthraquinone spacers was only obtained as the *trans* isomer, the yield of which was greatly improved by repeating the reaction at high (10 kbar) pressure. A supplementary study by Li and Becher^41^ replaced the anthraquinone moieties with further TTF units, which CPK modelling determined would allow for essential π–π donor–acceptor interactions only when the central bis-loop TTF unit was in the *cis*-configuration.

This proved to be true experimentally, and the corresponding handcuff catenane was obtained solely as the *cis*-isomer, whose *D*_2h_ symmetry was readily identifiable by ^1^H NMR spectroscopy. On the other hand, the anthraquinone *trans*-isomer possesses *D*_2_ symmetry and a strong temperature dependence is seen in the ^1^H NMR spectrum.^40^ Handcuff catenane formation was also explored with bis(macrocycles) incorporating 1,5- and 2,7-dioxynaphthalene spacing units.^42^ The 1,5-dioxynaphthalene containing bis(macrocycle) efficiently and exclusively formed the *trans*-isomer in 48% yield, whereas its constitutional isomer containing 2,7-dioxynaphthalene formed a mixture of handcuff isomers in a lower overall yield of 28%. CPK modelling showed only the *trans*-configuration of the bis(macrocycle) containing 1,5-dioxynaphthalene spacers can adopt an energetically favourable conformation for the self-assembly process; the 2,7-variant must twist into an energetically unfavourable state to achieve a suitable orientation for handcuff catenane formation.

Finally, Li and Becher^43^ examined the effect of modification to the cyclobis(paraquat-*p*-phenylene) component of their handcuff catenane systems by replacement of the phenylene linkers in the cyclophane with tetrafluorophenylene. Compared to the highly efficient *trans*-handcuff catenane formation for the 1,5-dioxynaphthalene spaced bis(macrocycle),^42^ the introduction of fluorine reduces the efficiency of the self-assembly process and also reverses the configurational selectivity. When both phenylene linkers are tetrafluorophenylene linkers, the *cis*-handcuff is the major product; when only one phenylene linker is fluorinated, the *trans*-isomer is still preferred over the *cis*-configuration in a 5 : 3 ratio, contrasting the exclusively *trans*-arrangement for unfluorinated cyclobis(paraquat-*p*-phenylene). In this case, π–π interactions between the threading species and the loops of the bis(macrocyclic) host become more relevant, as the electron deficient tetrafluorobenzene forms donor–acceptor stacking interactions with the electron rich 1,5-dioxynaphthalene units.

#### 3.1.2 Tethered-ring handcuff catenanes

In designing handcuff catenanes in which the two macrocycles of the bis(macrocyclic) component are separated spatially by a covalent tether that does not contain a useful chemical recognition site, some mechanism must be put in place to ensure the threading species passes through the macrocycles and remains there until the final macrocycle has been synthesised. To this effect, Sauvage and co-workers used an ion templating strategy in the self-assembly of a pseudo[3]rotaxane,^44,45^ where allylic ends of the two threaded species could be coupled together with simultaneous ring-closing metathesis (RCM) reactions (Fig. 7b). A bis(macrocycle) was designed with back-to-back chelating phenanthroline units that could take advantage of the templating effect of copper(i) cations to hold two phenanthroline threads within each of its cavities. The ends of these threads were held in close proximity, so the olefin metathesis reactions that form the final macrocycle proceeded efficiently, although no diastereomeric selectivity was reported. Despite the aromatic link between the two copper centres in the handcuff complex, no electronic communication between them was found by electrochemical analysis.

A very similar approach towards handcuff synthesis was realised by Beer and co-workers,^46^ who employed a chloride anion template to organise isophthalamide components in a suitable orientation for RCM reactions to afford a handcuff catenane (Fig. 7c). Elucidation of the single crystal structure of the handcuff revealed the importance of the chloride template by the orthogonal arrangement of isophthalamide recognition sites in the host bis(macrocycle) and guest macrocycle. In the solid state, bunching of the ethereal portions of the guest macrocycle and bis(macrocyclic) tether occurs creating a cryptand-like cavity, which the authors propose might serve as a useful cationic binding site. Overall, this might allow the handcuff catenane to function as an ion-pair receptor.

#### 3.1.3 Chemical topology in handcuff catenanes

**Fig. 8:**
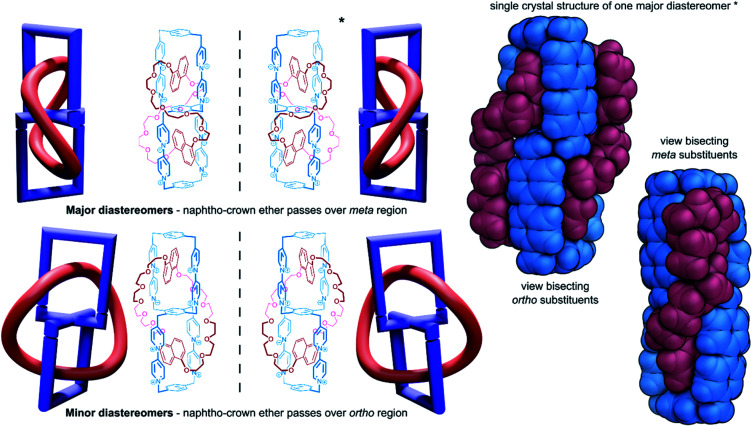
Chemical topology in handcuff catenanes governed by the large macrocycle orientation, with two views of the single crystal X-ray structure of the major diastereoisomer.

As with handcuff rotaxanes (see Section 2.2), handcuff catenanes also offer the possibility of topological isomerism as the components orient themselves around and through each other. Stoddart and co-workers have demonstrated such topological isomerism in the self-assembly of a (2)handcuff [2]catenane comprised of two fused cyclobis(paraquat-*p*-phenylene) cyclophanes through which a bis-1,5-dioxynaphtho[50]crown-14 macrocycle was threaded (Fig. 8).^47^ The synthesis of this handcuff catenane is unique in that the guest naphtho-macrocycle templates the formation of the bis(macrocyclic) host. Similar to the chiral handcuff rotaxane reported by Tokunaga,^21^ a pair of mirror-image enantiomers are formed depending on which direction the crown ether threads through the cavity of the second paraquat box of the bis(macrocycle). Unlike Tokunaga's system where the two rings of the bis(macrocycle) were fused orthogonally, in Stoddart's bis(macrocycle), the two cyclophane rings are fused about a phenyl ring, at 60° to each other, allowing the large macrocycle to wind about its host in two distinct paths. The naphtho-crown ether may pass over the central phenyl ring of the bis(macrocycle) through the small gap between the cyclophanes (designated the ‘*ortho*-region’), or through the large gap (the ‘*meta*-region’), creating a pair of topological diastereoisomers that cannot interconvert without the breaking of a covalent bond. Single crystal structure determination of the handcuff catenane ascertained that the major product of the synthesis was the *meta* isomer, which co-crystallised as an enantiomeric pair. Careful analysis of the handcuff in the solution state by NMR spectroscopy indicated the presence of another molecular species, whose identity was assumed to be the *ortho*-topological isomer. Interestingly, DFT calculations predict that in a vacuum the *ortho*-isomer is slightly favoured energetically, whereas under a solvation model the *meta*-isomer is thermodynamically preferred, matching the experimental outcome. Compared with handcuff rotaxanes, studies into the chemical topology offered by their catenane counterparts are much rarer. Many of the same opportunities for mechanoisomerism exist that may be explored in the future.

### 3.2 Cyclic handcuff catenanes

Cyclic bis[2]catenanes are mutually interlocked bis(macrocyclic) systems akin to two pairs of handcuffs that are arresting each other. Cyclic handcuff catenanes are topologically distinguished from linear (2)handcuff [2]catenanes, which are made from a total of three macrocycles instead of four. Here we note that related cyclic [2]rotaxane systems, or [c2] daisy chain rotaxanes,^48–50^ are the only remaining example of two-component doubly mechanically bonded structures, though in this case the absence of a bis(macrocyclic) species from their structure precludes them from classification as handcuff molecules. Preparation of cyclic bis[2]catenanes may follow many of the same design and construction principles seen already for handcuff rotaxanes and linear handcuff catenanes: *i.e.*, a bis(macrocycle) must form two mechanical bonds with a single guest species, though new synthetic avenues are also opened as a result of their homodimeric nature.

#### 3.2.1 Cyclic handcuff catenanes with simple tethers

**Fig. 9:**
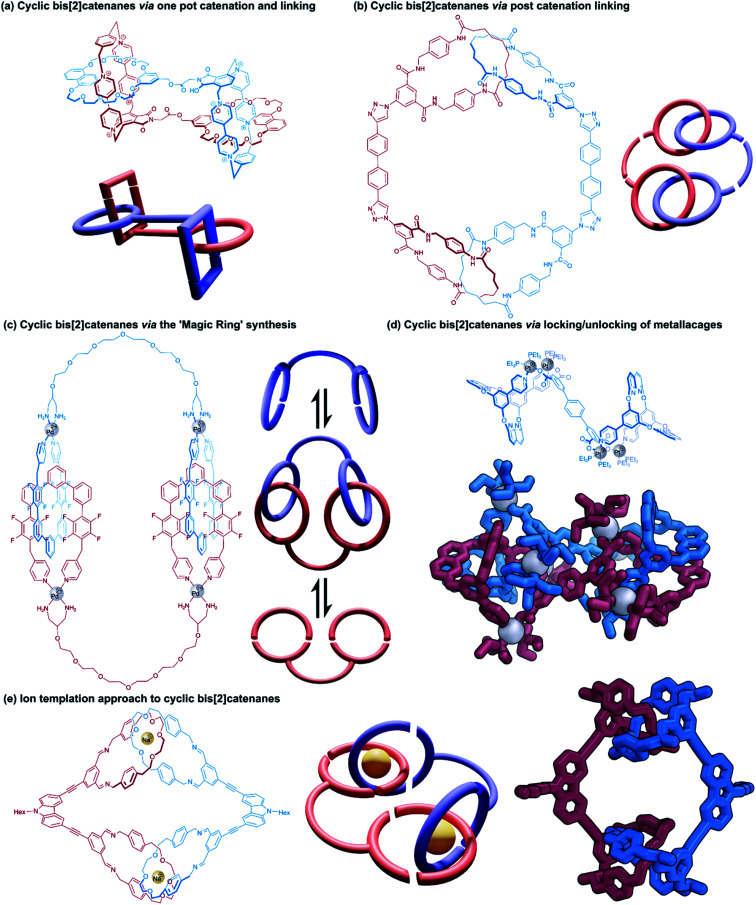
Cyclic bis[2]catenanes. (a) A one-pot approach with simultaneous catenation and linking; (b) post-catenation linking of [2]catenanes through azide–alkyne cycloadditions; (c) a ‘magic ring’ synthesis using the reversible ring locking/unlocking of Pd(ii) macrocycles; (d) using the reversible locking/unlocking of metallacages. Hydrogen atoms have been omitted on the representation of the single crystal X-ray structure; (e) templation around sodium ions to construct cyclic bis[2]catenates. The crystal structure of the reduced and methylated form without sodium ions. Hydrogen atoms have been omitted on the representation of the single crystal X-ray structure.

Some of the earliest examples of cyclic bis[2]catenanes that have been reported were side products in the attempted synthesis of pretzelanes,^9,10^ interlocked structures based upon [2]catenanes whose rings are also linked covalently. Vögtle and co-workers prepared a [2]catenane system from macrocycles that contained sulfonamide groups,^9^ offering a reactive site for further functionalisation. Alkylation with a suitable difunctional reagent did succeed in intramolecularly linking the two catenated rings, producing the pretzelane target, but also afforded a cyclic bis[2]catenane product where pairs of [2]catenanes are linked through reaction with two of the tethering molecules. Stoddart and co-workers also produced a cyclic bis[2]catenane as a side product of a pretzelane synthesis.^10^ An electron rich dioxynaphtho-crown ether was functionalised with a *p*-xylylene dibromide derivative that is capable of reacting to form the familiar electron deficient macrocycle cyclobis(paraquat-*p*-phenylene) cyclophane, when combined with its bis(paraquat-*p*-phenylene) precursor (Fig. 9a). The dioxynaphthalene group templates the formation of the cyclophane, so if the cyclisation occurs intramolecularly a pretzelane forms and intermolecular reactions lead to cyclic (or linear) catenane oligomers. Experimentally, the cyclic bis[2]catenane was favoured over the pretzelane with respective isolated yields of 20% and 14% for the two compounds. Stoddart's cyclic crown ether/paraquat cyclophane handcuff represents the only example of a bis[2]catenane where the bis(macrocyclic) component contains two different types of macrocycle.

Yano and co-workers have also reported several cyclic bis[2]catenanes as side products of poly[2]catenane synthesis, where [2]catenanes bearing reactive groups have been linked by further covalent coupling reactions.^51–53^ In some cases two complementary [2]catenanes were joined directly, for example, [2]catenanes containing pendent carboxylic acid groups were combined with [2]catenanes bearing pendent phenol or aniline moieties to form ester-bridged or amide-bridged polycatenanes and cyclic handcuffs.^53^ In another instance, a simple ditopic bisalkyne linker was employed as a means of linking [2]catenane monomers with pendent azide groups through a ‘click’ reaction (Fig. 9b).^51^

A targeted approach to cyclic handcuff catenane synthesis was reported by Fujita and co-workers as a method of producing exceptionally large ‘ultramacrocycles’.^54^ Taking advantage of the reversible ‘magic-ring’ catenation exhibited by palladium clipped macrocycles when exposed to polar solvents,^55^ a bis(macrocycle) was prepared that tethered two of these palladium macrocycles together (Fig. 9c). The addition of water to a DMSO solution of the palladium-bis(macrocycle) induced catenation of the coordination rings as cyclic handcuff dimers, confirmed by mass spectrometry and DOSY spectroscopy. The authors suggest that their technique of using reversible catenation overcomes some of the entropic disadvantages of uniting large components by providing a comparably large reaction centre. Due to the labile nature of the mechanical bonds in this system, this study represents an example of molecular handcuffs that can be unlocked and relocked at will.

Stang and co-workers have also recently reported a cyclic bis[2]catenane platinum metallacage that can be transformed between locked and unlocked topologies by guest exchange, concentration and solvent stimuli (Fig. 9d).^56^ The cyclic bis[2]catenane formed at much lower concentrations than homologous [2]catenane monomers of the untethered macrocycle, due to the synergistic effect of multiple catenation events.

Dynamic ring formation has also been used by Chiu and co-workers in the fabrication of cyclic bis[2]catenanes.^57^ The group had previously developed a [2]catenane synthesis that uses sodium ions to template the orthogonal alignment of two di(ethylene glycol) diamines which are then cyclised through condensation with isophthalaldehyde units.^58^ Chiu constructed a tetraaldehyde species that consisted of two rigid dialdehyde arms held at ∼90° to each other, to counteract the linearly functionalised catenane structural motif. Condensation of this tetraaldehyde with the protocatenane template gave a cyclic bis[2]catenane product (Fig. 9e), as well as cyclic trimers and tetramers.^57^ Reduction of the imine groups to amines locked the structure in place and allowed for purification of the handcuff.

#### 3.2.2 Cyclic handcuff catenanes with macrocyclic tethers

**Fig. 10:**
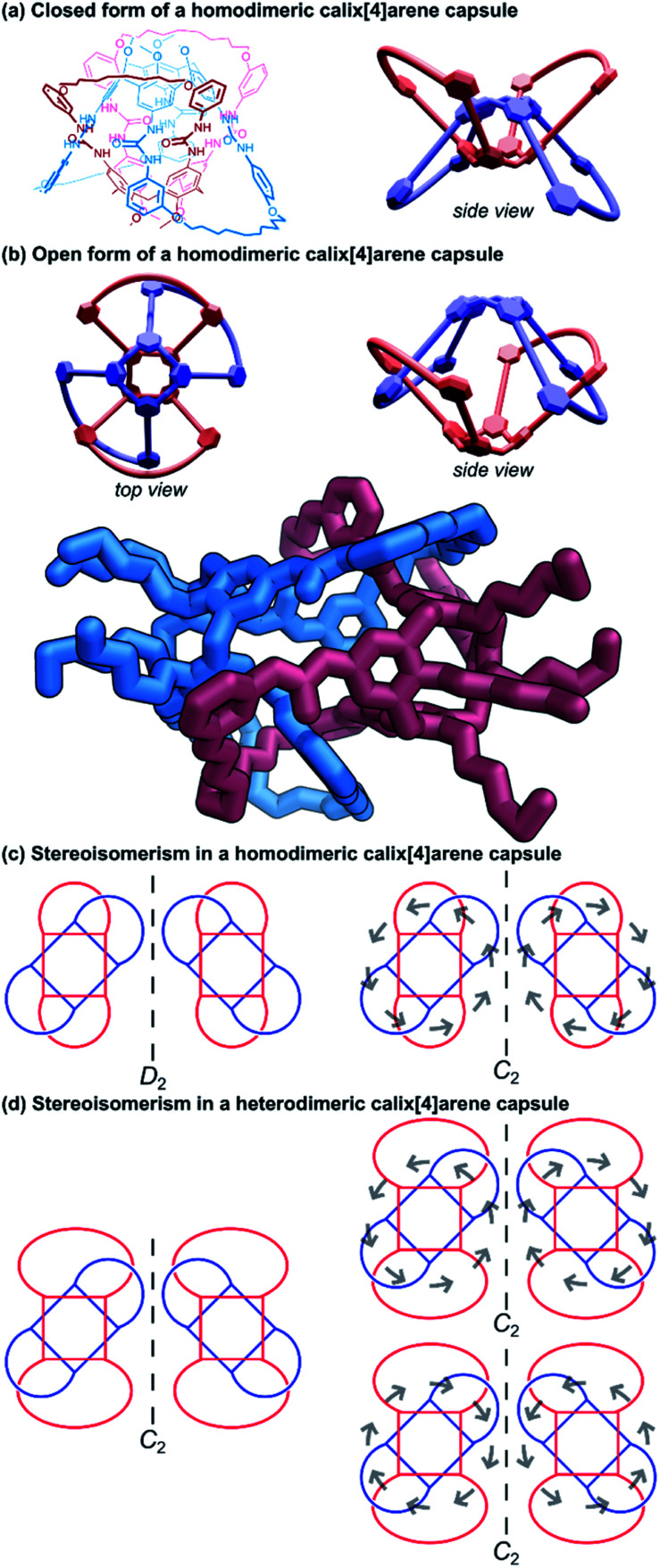
Cyclic (2)handcuff [2]catenanes synthesised *via* dimeric calix[4]arene capsules. (a) Chemical structure and schematic representation of such a handcuff in the closed form; (b) schematic representations and a crystal structure representation of the open form. Hydrogen atoms have been omitted on the representation of the single crystal X-ray structure; (c) stereoisomerism possible in a homodimeric handcuff capsule, with grey arrows indicating the direction of the hydrogen bonding in the urea belt; (d) stereoisomerism possible in a heterodimeric handcuff capsule, with grey arrows indicating the direction of the hydrogen bonding in the urea belt.

Another approach taken in the synthesis of cyclic bis[2]catenanes has been demonstrated by Böhmer and co-workers, based upon dimeric tetraurea calix[4]arene capsules.^59^ A calix[4]arene was functionalised on its wide rim with urea groups that lead into terminal octenyl residues. Under a solvent template, two such calix[4]arenes form hydrogen-bonded dimers and olefin metathesis was employed to form covalent connections between the octenyl residues. When the metathesis reaction unites adjacent residues of just one calix[4]arene, a cyclic bis[2]catenane product is obtained; though side products are possible if connections form between the two different calix[4]arene monomers in the dimeric capsule. Any *cis*/*trans* isomeric complications were removed by subsequent hydrogenation. Once mechanically bonded, the capsule can be closed (Fig. 10a) and opened (Fig. 10b) by changing solvent due to the relative stability of the hydrogen-bonded seam in solvents of differing polarity. This allows for the release and exchange of guest molecules.

The yield of the cyclic bis[2]catenane handcuff was greatly improved by first ensuring one of the calix[4]arenes was already connected as the bis-loop derivative before forming the handcuff, avoiding any possible cross-connection between the two monomeric units.^60^ This strategy also allowed for the formation of heterodimeric capsules, with different loop sizes.^61^ When the pairs of loops of the second calix[4]arene monomer are closed by the metathesis reaction, this may occur in either direction (that is to say a particular thread may react with its clockwise or anticlockwise neighbour). In the homodimeric handcuff capsule, this leads to an enantiomeric pair of topological isomers of the resulting bis[2]catenane with *D*_2_ point group symmetry. However, the point group symmetry is reduced to *C*_2_ when the cyclodirectionality of the hydrogen-bonded urea belt (which is kinetically stable) is considered (Fig. 10c). The parent heterodimeric handcuff capsule possess *C*_2_ point group symmetry. The cyclodirectionality of the hydrogen-bonded urea here adds an independent element of chirality but does not change the point group and so two diastereomeric pairs of enantiomers are observed (Fig. 10d).

Böhmer and co-workers were able to chromatographically resolve the two enantiomers of various capsules with a chiral stationary phase. The reversible opening of the capsule combined with the chirality might lead to useful applications of these handcuff molecules in the discrimination of chiral guest moieties.

Chas and Ballester^62^ built upon Böhmer's earlier calix[4]arene capsule work and created heterodimeric handcuff capsules built from interlocked calix[4]arene and calix[4]pyrrole hemispheres. The substitution of one of the calix[4]arenes with a calix[4]pyrrole provides different functionality to each interior pole of the capsule. Amine oxide guest molecules were found to interact strongly with the capsule; the pyrrole portion forms hydrogen bonds with the *N*-oxide and the calixarene provides stabilising C–H–π interactions. Stabilisation of particularly reactive *N*-oxide guests was also possible by confinement within the handcuff capsule.^63^ The polarised inner space also permitted co-encapsulation of ion pairs; experiments with both tetramethylammonium chloride and tetraethylphosphonium chloride salts showed complete encapsulation of both ions of the salt.^62^ The co-encapsulation of such guests might allow for the possibility of using this architecture as a vessel for certain chemical reactions. More recent work performed by Ballester and co-workers^64^ examined the use of the calix[4]arene/calix[4]pyrrole handcuff to encapsulate a series of alkylated *N*,*N*-dimethylamine *N*-oxide guests where the non-methyl alkyl group varied in length from C1 to C10. Smaller *N*-oxides were co-encapsulated with a solvent molecule which would occupy the calixarene hemisphere; larger *N*-oxide molecules occupied the entire cavity by themselves, with the alkyl chain undergoing severe coiling to fit into the capsule. Competitive addition of shorter *N*-oxides to capsules hosting longer ones saw the complete replacement of the longer guest with the shorter one, even if it also meant entropic costs associated with encapsulating a solvent molecule as well, revealing the capsule's ability to stabilise sterically demanding *gauche* states of coiled aliphatic chains. Furthermore, the advantages of the handcuff topology were established by comparing host properties against an analogous non-interlocked capsule. The largest guest that could be trapped without the supporting mechanical bonds bore just a C5 chain, thus, the mechanical bonds helped stabilise against the thermodynamic instability of the multiple *gauche* interactions present for the convoluted aliphatic chains.

## 4. Higher handcuff architectures

Finally, we turn our attention to molecular handcuffs of a higher order; species that possess not only the doubly interlocked handcuff motif described thus far but carry further complexity in the form of additional mechanical bonds. As an example, a molecular structure comprising a four ring system that is able to lock on to a guest molecule with four separate ‘limbs’ would be analogous to a harness style restraint that simultaneously binds the wrists and ankles. This arrangement would be called a (4)handcuff [2]rotaxane under our nomenclature, since the hosting component has four occupied rings.

Developments in the field of mechanically interlocked chemistry have allowed for the construction of many intricate molecular architectures. Within this section we describe supramolecular bundles, pseudo[4]catenanes, as well as examples of handcuff systems that involve more than just two components. The limit to the complexity that can be achieved by molecular handcuffing is still far from becoming realised.

### 4.1 Threefold interlocked handcuff structures

Beginning our exploration of higher order handcuffs are structures that involve expansion of the bis(macrocyclic) component to a three-loop tris(macrocycle). The arrangement of the three macrocycles within the host offers a new avenue of topological considerations: the three rings may be coplanar or orthogonal to a common plane (or perhaps neither), likewise the three rings might be arranged linearly or branching from a central point. These differences in host topology can have a profound effect on the structure of the guest molecule, which could thread all rings from the same side, as is the case for supramolecular bundles, or pass through the rings in sequence from one end to the other, like a braid. The relationship between tris(macrocyclic) systems and (2)handcuffs should be apparent when considering the threading of the guest component. If during association of the two components the guest fails to thread through the third macrocycle, the resulting supramolecule would still bear all the hallmarks of a traditional (2)handcuff. As such, we reason that whether the third macrocycle is threaded or not, the resulting structure should be considered part of the handcuff family of supramolecules.

#### 4.1.1 Handcuff rotaxanes with three mechanical bonds – (3)handcuff [2]rotaxanes

The first host–guest inclusion complex using a tris(macrocyclic) host and trivalent guest was reported by Stoddart and co-workers.^65^ A triphenylene core was used to fuse three benzo[24]crown-8 macrocycles and a trifurcated guest was made from three dibenzylammonium cations linked in the *meta* positions around a central benzene core. The resulting 1 : 1 assembly of these components was termed a ‘supramolecular bundle’. The collective stabilising π–π interactions between the cores of the two components as well as hydrogen bonding between the crown ether and ammonium recognition sites meant that the compound retained its integrity in the solution phase, despite the lack of stoppering groups, although dethreading could be stimulated by the addition of base. Rotaxane variants based on the same tritopic recognition motif were later constructed by post-assembly stoppering^66^ and by templating formation of the tris(macrocycle) with the trisammonium guest in a triple ring closing metathesis reaction.^67^

**Fig. 11:**
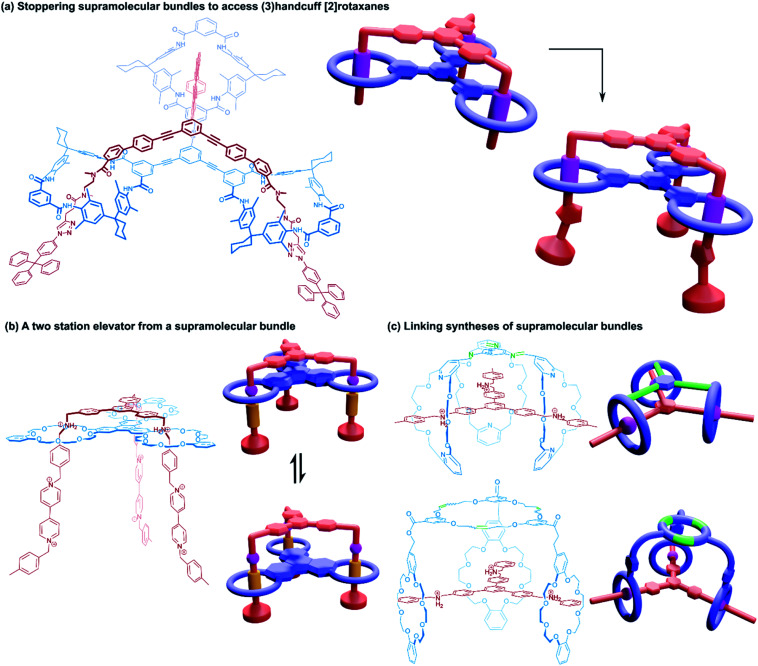
(3)Handcuff [2]rotaxanes. (a) The stoppering of a supramolecular bundle made from a tris(tetralactam) host *via* azide–alkyne cycloadditions to give a (3)handcuff [2]rotaxane; (b) a two station molecular elevator with a platform that moves between viologen and dibenzylammonium stations; (c) linking synthesis of supramolecular bundles using olefin metathesis and imine formation.

A study into the kinetics of the multivalent bundle threading was also performed with a related guest molecule that contained dicationic viologen units instead of the dibenzylammonium recognition sites.^68^ Threading of two of the viologen arms into the receptor proceeded almost instantly, whereas the third threading event was considerably slower, eventually equilibrating to the fully threaded complex after ten days. Schalley and co-workers also evaluated the thermodynamic threading behaviour of trivalent pseudorotaxanes constructed from a tris(tetralactam) host and a trifurcated axle with three bisamide arms using NMR and ITC experiments.^69^ The second binding event was found to exhibit a slightly positive cooperativity, the third binding event, however, was non-cooperative due to unfavourable strain, perhaps due to a slight mismatch in the size of the receptor and guest. The preorganisation of the host and guest components still ultimately ensures formation of the completely threaded complex, and after stoppering with copper-catalysed azide–alkyne Huisgen cycloaddition reactions gives the (3)handcuff [2]rotaxane (Fig. 11a).

Stoddart and co-workers also constructed (3)handcuff [2]rotaxane supramolecular bundles with axles whose arms contained both dibenzylammonium and viologen stations (Fig. 11b).^70,71^ These elevators could be operated as bistable rotaxanes. Through the addition of base the dibenzylammonium sites are deprotonated and the tris(macrocyclic) ‘platform’ moves along the axle to the viologen stations, travelling a distance of 0.7 nm. Subsequent protonation of the ammonium stations by acidification restabilised the platform in its upper position. The authors estimate that the downward force exerted by the tris(macrocyclic) platform is around 200 pN, roughly one order of magnitude larger than forces provided by linear motors such as the muscle protein myosin.^72^ Interestingly, the motion of the host appears to follow a three-step pathway rather than moving smoothly in a concerted process. Schalley and co-workers also constructed a switchable (3)handcuff [2]rotaxane elevator based upon the tris(tetralactam) host and a trifurcated axle with bisamide and triazole stations.^69^ The addition of chloride to the rotaxane caused a shift in binding from the bisamide to triazole sites, which can be completely reversed by precipitation of the chloride using sodium tetraphenylborate.

**Fig. 12:**
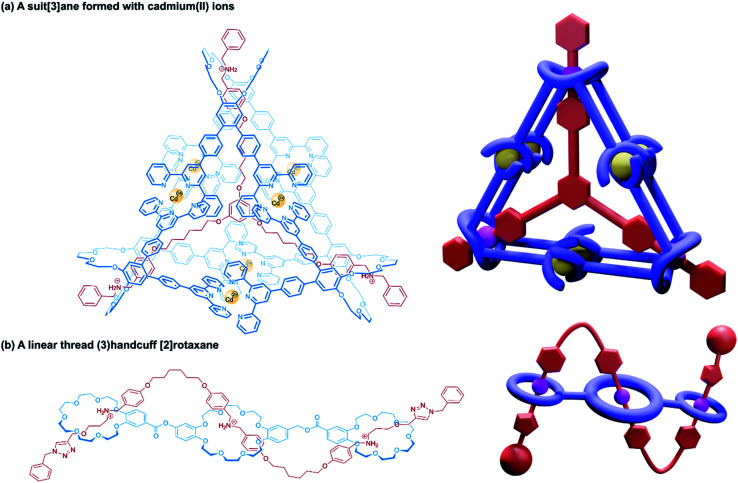
Other approaches to (3)handcuff [2]rotaxanes. (a) A suit[3]ane formed from the coordination of terpyridine groups to cadmium(ii) ions; (b) a braided (3)handcuff [2]rotaxane architecture.

Alternative approaches to (3)handcuff [2]rotaxane synthesis have also been reported that make use of a linking approach (see Section 2.1.1) to covalently join three macrocycles together in a pseudo[4]rotaxane. In a collaboration between Stoddart and Grubbs,^73^ the familiar 1,3,5-tris(dibenzylammonium) axle self-assembled with three equivalents of a dibenzo[24]crown-8 derivative that bore two olefinic side arms. A simultaneous olefin metathesis reaction was able to link the three threaded macrocycles, forming a cyclic trimer of the crown ether monomers and yielding the (3)handcuff [2]rotaxane (Fig. 11c). In the absence of the template this unique cyclisation could not occur, and no trimers were formed. A dynamic covalent strategy in tandem with the tritopic template approach was used to link together formyl appended dipyrido[24]crown-8 macrocycles through reaction with 1,3,5-triaminobenzene to form imine bonds (Fig. 11c).^74^ The short bridging unit between the three macrocycles constrained the system rigidly enough to provide mechanical bonding, even without the need for conventional stoppers, akin to suitanes (see Section 2.2). Indeed, a suit[3]ane was also synthesised through the reaction of two 1,3,5-triaminobenzene caps with three dipyrido[24]crown-8 rings that were functionalised on both pyridine units with formyl moieties in the presence of the trisammonium template. Chan and co-workers also created a suit[3]ane by linking together the three macrocyclic monomers with coordination bonds.^75^ A dibenzo[24]crown-8 macrocycle was functionalised with four terpyridine groups, two for each of the macrocycle's benzene rings. Coordination of these ligands with cadmium(ii) ions generated a metalloprism ‘suit’ that could encapsulate a tris(ammonium) guest molecule, connected to a central 1,3,5-substituted benzene core by flexible aliphatic chains (Fig. 12a). Encapsulation of the guest component further stabilised the complex.

So far all the axle components of the (3)handcuff [2]rotaxanes described have had a radially branched structure, where identical recognition sites extend as spokes from a central unit. In contrast, a linear threadlike component with three ammonium recognition sites was used by Tian and co-workers in the construction of a handcuff rotaxane with a braided topology.^76^ A tris(macrocycle) with a central DB24C8 unit and smaller flanking benzo[21]crown-7 macrocycles was mixed with the flexible tris(ammonium) axle, which self-assembled into the fully threaded pseudo[2]rotaxane, with subsequent introduction of phenyl stoppers (Fig. 12b). Since the phenyl rings of the axle are too large to pass through the cavity of the benzo[21]crown-7 macrocycles, the mechanism of threading cannot proceed *via* a single leading end passing in turn through all three macrocycles, but the central DB24C8 must at least be threaded ahead of one of the smaller outer macrocycles in order to produce the (3)handcuff.

**Fig. 13:**
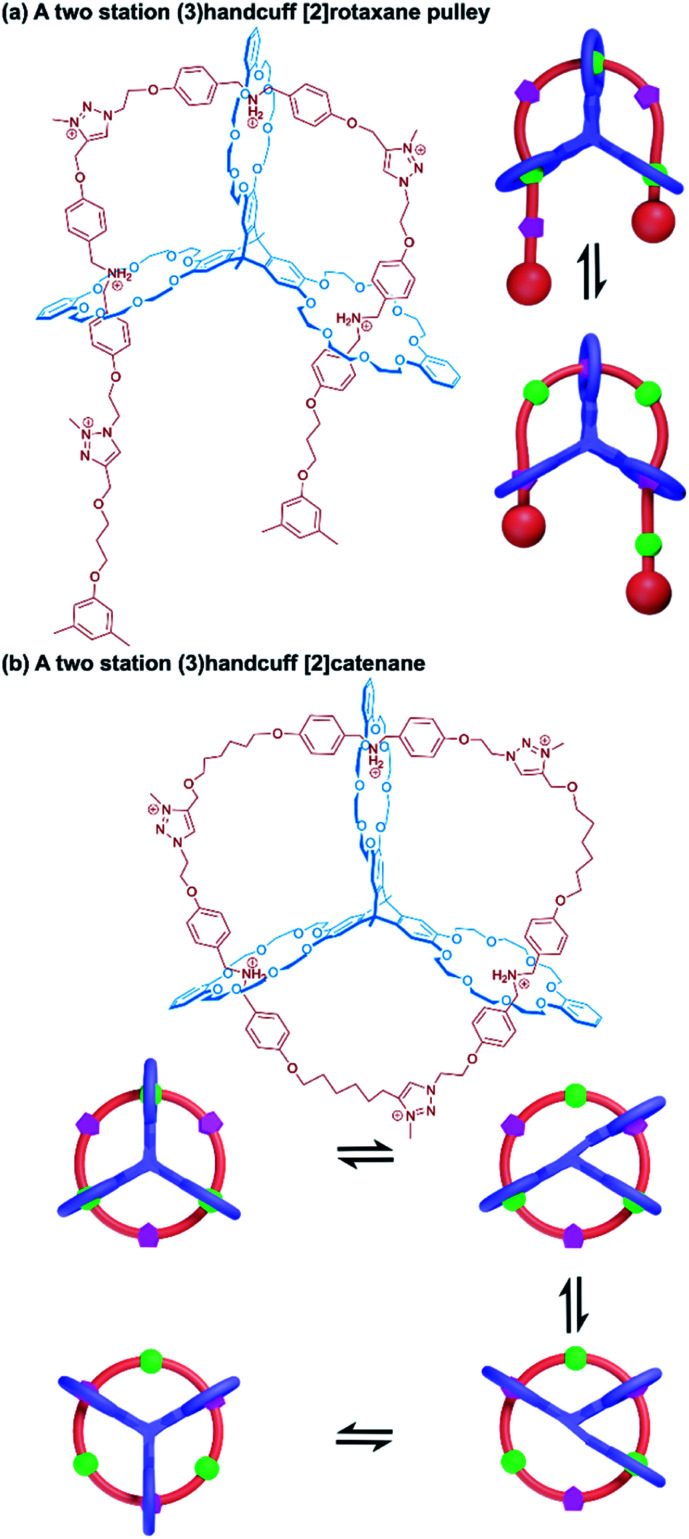
Two station (3)handcuffs based upon a tris(macrocycle) derived from a triptycene core. (a) A [2]rotaxane pulley with dibenzylammonium and triazolium stations; (b) stepwise motion of the macrocycles in a (3)handcuff [2]catenane between dibenzylammonium and triazolium stations.

A linear thread was also used by Meng and Chen to fabricate a molecular pulley based upon a triply interlocked [2]rotaxane.^77^ A triptycene core connected three DB24C8 macrocycles so that the planes of the macrocycles were rotated 120° with respect to each other. An axle molecule made of alternating dibenzylammonium and triazole stations, stoppered at one end, was able to thread sequentially through the host's three rings, following the inchworm mechanism described earlier.^19^ Once fully associated the axle was stoppered on its leading end (introducing a third triazole group as part of the reaction) to give a (3)handcuff [2]rotaxane. Methylation of the triazole stations created cationic triazolium recognition sites (Fig. 13a). The thread of the system could be pulled through by deprotonation of the ammonium sites, causing the macrocycles to favour the triazolium stations and subsequently retracted by reprotonating, similar to the motion of a rope about a pulley. The unique pulley geometry combines the linear translocation of a [2]rotaxane with the rotary motion typical of a [2]catenane.

#### 4.1.2 Handcuff catenanes with three mechanical bonds – (3)handcuff [2]catenanes

The work of the Chen group has also extended to producing triply interlocked catenane structures based upon the triptycene tris(macrocycle) described above. Their first (3)handcuff [2]catenane was synthesised from a triple olefin metathesis reaction from a pseudo[4]rotaxane precursor.^78^ The triptycene tris(macrocycle) was threaded through each cavity by a dibenzylammonium thread functionalised with terminal olefin groups. The tris(macrocycle) positioned the olefin termini of the threads in close proximity, facilitating the ring-closing metathesis reaction and any isomeric complications were removed by hydrogenation. This method of handcuff catenane synthesis is very similar in approach to the (2)handcuff [2]catenane work of Sauvage^44,45^ and Beer^46^ outlined in Section 3.1.2, where the large guest macrocycle is formed by multiple olefin metathesis reactions. The crystal structure of the (3)handcuff [2]catenane showed three large cavities formed by the space between the triptycene core of the host and the large guest macrocycle. Later work demonstrated it was also possible to produce the same (3)handcuff [2]catenane architecture by starting with an olefin-terminated tris(benzylammonium) strand.^79^ Interestingly, if this strand was already conjoined as a macrocycle and later mixed with the tris(macrocyclic) host, a reversible ring-opening ring-closing metathesis reaction could be stimulated by the addition of Grubbs II catalyst, forming the triply catenated product in high yield. A chiral variation was also reported,^80^ where the tris(macrocycle) was modified with (*R*)-1,1′-binaphthyl moieties. Post-synthetic *N*-acylation of the tris(ammonium) thread created stopper units that limited rotation of the large macrocycle. Compared to the unthreaded host, the guest's presence greatly reduced the Cotton effect of the binaphthyl chromophore at 241 nm, but increased the effect at 248 nm, possibly indicative of chirality transfer to the large threaded macrocycle.

More recently, Chen and co-workers developed a (3)handcuff [2]catenane from a pyrazine extended version of their triptycene tris(crown ether) and a thread made from alternating dibenzylammonium and methyltriazolium stations.^81^ Familiar to us from related systems, deprotonation of the ammonium sites caused rotation of the thread so that the triazolium stations associated with the cavities of the tris(macrocyclic) host, which could be reversed by reprotonation. The switching between these two states also corresponded with a change in emission intensity from the central quinoxaline fluorophore. A detailed investigation into the relative motion of the two components revealed that a stepwise motion was generated by the appropriate stimulus; with the crown ether moieties migrating one at a time to their new stations through a series of four stable states (Fig. 13b). This stepwise motion also supports the one-ring-at-a-time observation of motion in the molecular elevator described in the previous section.^71^

### 4.2 Fourfold interlocked handcuff structures

Increasing the ring count of the host component from three to four boosts the topological possibilities of the resulting handcuff systems as new connective opportunities are available for arranging the rings before even considering their three-dimensional orientations. Fortunately for our discussion, the difficulties of synthesising such highly intricate topological systems have limited the structures of (4)handcuff systems presently realised to those that contain a tetrakis(macrocyclic) host with four rings branching symmetrically from a central nucleus.

#### 4.2.1 Handcuff rotaxanes with four mechanical bonds – (4)handcuff [2]rotaxanes

**Fig. 14:**
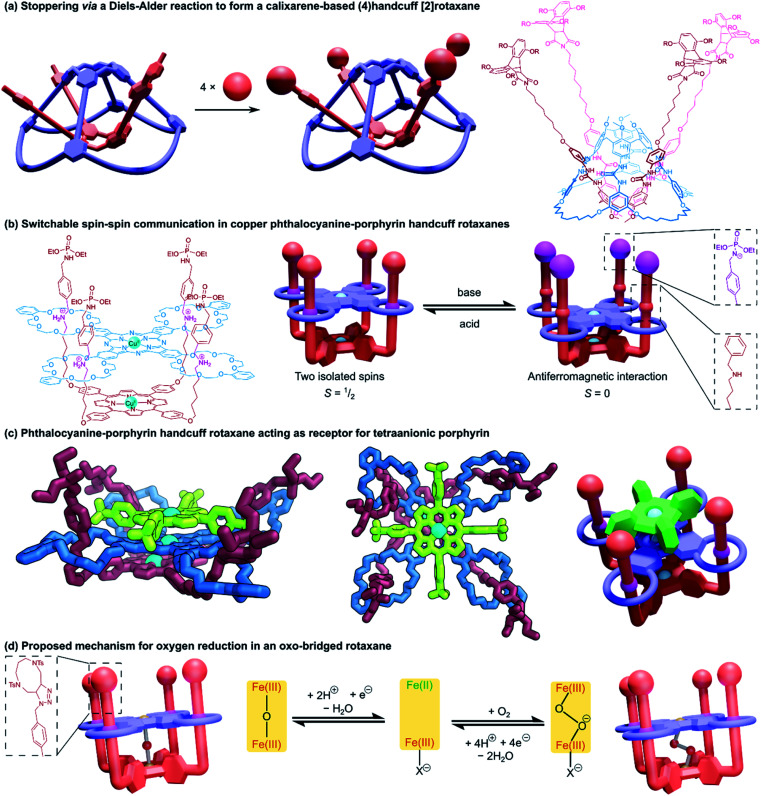
Structures of (4)handcuff [2]rotaxanes. (a) Diels–Alder reactions between maleimide and anthracene units to stopper a dimeric calix[4]arene capsule; (b) Addition of acid or base to a copper phthalocyanine–porphyrin handcuff rotaxane moves the handcuff position along the axle and controls the spin–spin communication between the metal centres; (c) a phthalocyanine–porphyrin handcuff rotaxane acting as a receptor for a third tetraanionic porphyrin. Hydrogen atoms have been omitted on the two views of the single crystal X-ray structure; (d) proposed mechanism for oxygen reduction in an oxo-bridged handcuff rotaxane.

Quadruply interlocked [2]rotaxanes were first reported by Böhmer and co-workers^82,83^ who made use of the well-defined hydrogen-bonded arrangement between two urea functionalised calix[4]arenes (see Section 3.2.2) to organise the components for mechanical bonding. In the first instance,^82^ a tetraloop calixarene was prepared by olefin metathesis of an tetra-bis(alkenyl) functionalised calix[4]arene,^84^ where adjacent alkenyl arms were coupled to produce the four new macrocycles. When mixed with a second calix[4]arene derivative carrying four pendent alkylmaleimide arms in apolar solvents, heterodimeric capsules of the two calixarenes formed, with the alkylmaleimide arms threaded through each of the four loops of the host component (Fig. 14a). This pseudo(4)handcuff [2]rotaxane could then be stoppered through a Diels–Alder reaction between the maleimide groups and 1,4,5,8-tetraalkoxyanthracene. In the other instance,^83^ the tetraloop host was created by ring closing around the four armed guest calixarene in a heterodimeric capsule of the two calix[4]arenes. This alternative pathway allowed for the use of a shorter axle component and smaller threaded macrocycles. The more tightly knitted (4)handcuff [2]rotaxane was stable in solvents that would typically break the hydrogen bonded seam between the two calixarene hemispheres and exchange of solvent molecules entrapped within the capsule was completely suppressed. As such the authors compared this fourfold rotaxane to a mechanically bonded equivalent of a hemicarcerand.

Tanaka and co-workers employed mechanical bonding as a method of cofacially stacking dimers of porphyrin and phthalocyanine chromophores.^85^ A phthalocyanine unit with four peripheral DB24C8 macrocycles and a porphyrin substituted with four benzylammonium arms formed a pseudorotaxane complex that was locked with the introduction of phosphoramidate stopper groups to the threaded arms of the porphyrin (Fig. 14b). Both porphyrinic centres were then metallated with copper(ii). In the fully protonated (4)handcuff [2]rotaxane, the two copper centres do not interact and the EPR spectrum of the complex shows two isolated doublet (*S* = 1/2) spins. When deprotonated by strong base, the two copper centres move closer together, allowing an antiferromagnetic (*S* = 0) interaction between the spins. The switchable spin–spin communication afforded by this mechanically interlocked architecture was recognised as valuable progress towards spintronic nanomachines.

Subsequent work exploited the size and charge complementarity between the porphyrin–phthalocyanine (4)handcuff and a tetraanionic porphyrin to form a three membered columnar array,^86^ sandwiching the tetrakis(macrocyclic) phthalocyanine between the two porphyrin species (Fig. 14c). The identity of the metal centres of each member of the (4)handcuff assembly could be programmed, either by premetallating the components prior to rotaxane self-assembly,^87^ or by site specific metallation of either component.^88^ The phthalocyanine host selected for manganese(ii) ions, the porphyrin guest showed selectivity for iron(ii) and nickel(ii) ions. Complexation of the rotaxane to the tetraanionic porphyrin expanded the heterometallic array to a third metal ion. The spin–spin communication of paramagnetic copper(ii) ions confined within the central phthalocyanine moiety could be programmed by the identity of metal centres in the surrounding porphyrins.^89^ Similarly, the electrochemical properties of the (4)handcuff [2]rotaxane were affected by the charge density of the metal within the electrostatically bonded porphyrin.^90^

The close-stacked but flexible arrangement of metal centres within the porphyrin–phthalocyanine could also be exploited for catalytic purposes. Metallation with iron(ii) followed by aerobic oxidation afforded a μ-oxo-bridged complex that showed efficient catalytic reduction of oxygen to water (Fig. 14d).^91^ The structural adaptability provided by the mechanical bonds suitably accommodated peroxide and superoxide intermediates in the proposed catalytic pathway. A μ-nitrido-bridged diiron version of the (4)handcuff showed oxidative properties towards methane and ethane in the presence of hydrogen peroxide.^92^ The catalytic potency was enhanced by the addition of a tetraanionic porphyrin cofactor, which caused electronic perturbation of the metallic active sites. For greater detail, an in-depth review of their fourfold rotaxane work was recently published by Yamada and Tanaka within the context of face-to-face assemblies of porphyrinoids.^93^

A related (4)handcuff [2]rotaxane has been reported^94^ that combines a resorcinarene-based cavitand with four crown ether macrocyclic appendages with a porphyrin decorated with four imidazolium arms. Similar in architecture to Tanaka's (4)handcuff [2]rotaxanes, the authors compared the cavitand to a “bowl” and the porphyrin component to the lid of that bowl.

#### 4.2.2 Handcuff catenanes with tetrakis(macrocyclic) hosts – (4)handcuff [2]catenanes

Catenane structures based upon the tetraloop calixarene described above have also been synthesised by the Böhmer group.^95–97^ The assembly of heterodimeric capsules between the tetraloop calix[4]arene and calix[4]arenes substituted with four terminal olefin groups allowed for a ring closing metathesis reaction to occur between the adjacent alkenyl residues, forming a multiply catenated architecture.

**Fig. 15:**
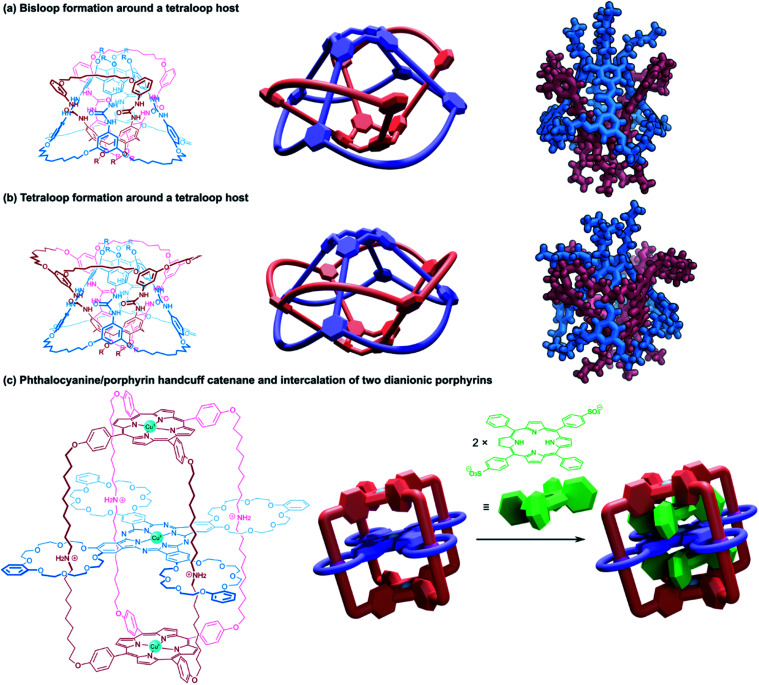
Structures of (4)handcuff [2]catenanes. (a) Bisloop formation around a tetraloop host synthesised *via* olefin metathesis/hydrogenation, with a view of the single crystal X-ray structure; (b) tetraloop formation around a tetraloop host synthesised *via* olefin metathesis/hydrogenation, with a view of the single crystal X-ray structure; (c) a quadruply interlocked phthalocyanine/porphyrin handcuff into which two dianionic porphyrins could intercalate.

The two newly created macrocyclic rings each formed mechanical bonds with two of the loops in the tetrakis(macrocyclic) calixarene template, resulting in a cyclic catenane topology (Fig. 15a). The structure resembles a pair of (2)handcuff [2]catenanes with separate covalent connections between the bis(macrocyclic) components and threaded guests, or rather, the structure could be reduced to two discrete (2)handcuff [2]catenane molecules by cutting along a mirror plane perpendicular to the interlocked rings. A crystal structure confirmed the interlocked arrangement of the two calixarene hemispheres.^96^ Catenated homodimers of the tetraloop calixarene were also synthesised by starting with a tetra(bis)alkenyl guest. A total of eight catenated rings can be seen in the crystal structure of the compound (Fig. 15b).^95^ Starting with the open-loop form of the tetraloop species was also considered as a synthetic route to both these structures, although it proved much more effective to form the tetrakis(macrocyclic) host first due to limiting the number of ‘wrong’ connections between the terminal olefinic side-chains during the ring-closing metathesis reaction. As with the rotaxane analogue above, the tight-knit structure of the capsule provided a cavity for (almost) permanent guest inclusion; the strength of the inclusion could be modulated by the number of catenated rings, and by their length.^96^

Tanaka and co-workers have also reported a multiply interlocked catenane derived from their porphyrin–phthalocyanine model.^98^ A pseudorotaxane consisting of the tetrakis(macrocyclic) phthalocyanine threaded with a porphyrin substituted with long alkylammonium arms that terminated in benzaldehyde residues underwent reaction with pyrrole, creating a new porphyrin cap from the benzaldehyde arms and locking the structure as a catenane (Fig. 15c). The movement of the phthalocyanine component along the alkylammonium arms allowed for the invasion of two dianionic porphyrins, which could occupy the space between the phthalocyanine platform and the porphyrin end caps. As a trinuclear copper(ii) complex, the spin–spin communication could be modulated by pH, or switched off by the intercalation of dianionic porphyrins.

### 4.3 Three component handcuffs – (*n*)handcuff [3]rotaxanes

**Fig. 16:**
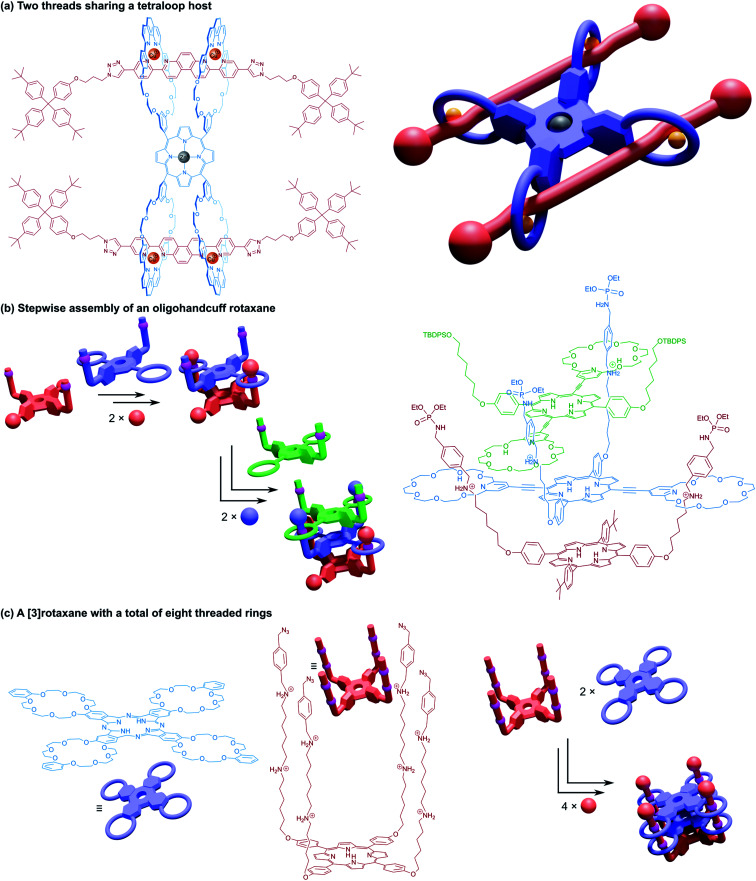
Structures of multiple component handcuff rotaxanes. (a) Two threads sharing a tetraloop host held in place with copper(i) phenanthroline coordination before stoppering; (b) stepwise assembly and stoppering of an oligohandcuff rotaxane; (c) two tetrakis(macrocyclic) hosts threaded onto a single tetrafurcated axle.

We conclude our discussion of higher order handcuff architectures with a few examples of handcuff rotaxanes that contain more than two distinct components that are interlocked by the handcuff motif *i.e.*, each threadlike component must form at least two mechanical bonds with its host. Sauvage and co-workers synthesised a tetrakis(macrocyclic) porphyrin with each loop containing a 1,10-phenanthroline group.^99^ An axle molecule was designed with a central bis-3,8-(*o*-pyridyl)-4,7-phenanthroline fragment, such that the gathering and threading effect of copper(i) would associate each axle molecule with two of the macrocycles of the porphyrin host. The pseudo[3]rotaxane underwent reaction with encumbering stopper groups to generate a [3]rotaxane species that resembles two (2)handcuff [2]rotaxanes that are connected by linking the tether between the bis(macrocyclic) host of two separate molecules (Fig. 16a).

Tanaka and co-workers published a method of repetitive stepwise handcuff rotaxane formation that begins with a *trans*-A_2_B_2_ porphyrin functionalised with two benzylammonium arms (Fig. 16b).^100^ A *trans*-bis(macrocylic) porphyrin could then be threaded on to this axle as a (2)handcuff [2]rotaxane after stoppering. The remaining *meso* positions of the bis(macrocyclic) porphyrin were then converted into new benzylammonium arms and the threading-stoppering sequence of reactions could be repeated to add more porphyrin layers. In principle, this ‘daisy-chain’ strategy of oligomeric (2)handcuff [2]rotaxane synthesis could be extended to any number of porphyrin layers. The control of sequence offered by this approach for a similar metalloporphyrin assembly presents great opportunity to develop molecular wires and unique spintronic devices.

Finally, we turn our attention to a handcuff [3]rotaxane synthesised by the Tanaka group in 2016.^101^ Their fourfold rotaxane approach was repeated with a porphyrin axle, decorated in each of its *meso* positions with long arms containing two alkylammonium stations (Fig. 16c). This axle was able to assemble into a 1 : 2 complex with two equivalents of their tetra(macrocyclic) phthalocyanine host; the first phthalocyanine moving along the axle to the second set of alkylammonium stations to allow a second phthalocyanine to thread onto the axle before stoppering. Electrochemical measurements of the [3]rotaxane indicated substantial electronic interaction between the components, especially the two phthalocyanine hosts. When both phthalocyanines were added as their copper(ii) complexes, the trimeric assembly displayed an antiferromagnetic interaction in its protonated form, contrasting the spin–spin interaction of the analogous dimeric stack.^85^ This three component architecture comprising a total of eight threaded rings represents the most complicated molecular handcuff species we have encountered to date, and likely one of the most intricate mechanically bonded systems currently achieved.

## 5. Conclusions

In this article we have attempted to bring together the variety of mechanically interlocked molecules that employ the use of a molecular handcuff motif. One issue we have encountered in doing so, is the variation and breadth of terminology that has been used to discuss these topologically related structures. The diffuse terminology that has been employed in the field has perhaps obscured the commonalities in approaches that have been used across these molecular systems. We have attempted to bring together systems which are conceptually associated, and in doing so have introduced a new nomenclature that can be employed to compare and contrast this range of structures. We anticipate that our new terminology will aid researchers to describe their structures more readily, and to identify related structures for comparison.

Many successful strategies have been developed to synthesise molecular handcuffs that build upon traditional mechanically interlocked techniques. The advancement of handcuffed structures has led to unique examples of topological chirality,^102^ as well as unique control of relative motion, seen in examples of elevators^35,69–71^ and pulleys.^77^ The precise but flexible arrangement of molecular fragments offered by handcuff rotaxane formation has enabled unprecedented investigation of molecular interactions, whether through communication between redox active chromophores,^35,38,39^ or through programmable spin–spin interactions across nearby metal cations.^85,89,98,101^ These studies illustrate the exceptional power of the handcuff scaffold as a tool for positioning chemical entities, where the adaptable, flexible nature of the mechanical bond prevails over the more restricting covalent counterpart.

Another feature of reviewing the variety of handcuff structures is to draw out synergies between different systems. We hope that readers of this article will see new approaches to the expansion of available handcuff architectures. Indeed, it becomes apparent that the use of handcuffs to assemble new molecular constructs is perhaps only limited by our imagination. As such, we wish to stimulate our readers' creativity and encourage researchers within this field to explore the use of handcuffs in their own mechanically interlocked designs.

## Author contributions

M. T., N. J. A., and A. W.-L., contributed equally to the writing of this manuscript. Figures were created by M. T., N. J. A., and A. W.-T. with direction from all authors. All authors conceived the idea and created the outline with equal contributions and the text was compiled and refined by N. P. with input and direction from B. S. P. and N. R. C.

## Conflicts of interest

There are no conflicts to declare.

## Supplementary Material
